# Deep immunophenotyping reveals clinically distinct cellular states and ecosystems in large-scale colorectal cancer

**DOI:** 10.1038/s42003-023-05117-1

**Published:** 2023-07-27

**Authors:** Si Li, Tao Pan, Gang Xu, Yueying Gao, Ya Zhang, Qi Xu, Jiwei Pan, Weiwei Zhou, Juan Xu, Qifu Li, Yongsheng Li

**Affiliations:** 1 Key Laboratory of Tropical Translational Medicine of Ministry of Education, College of Biomedical Information and Engineering, Hainan Women and Children’s Medical Center, Hainan Medical University, Haikou, 571199 China; 2 School of Interdisciplinary Medicine and Engineering, Harbin Medical University, Harbin, 150081 China; 3 College of Bioinformatics Science and Technology, Harbin Medical University, Harbin, 150081 China; 4 The First Affiliated Hospital, Hainan Medical University, Haikou, 571199 China

**Keywords:** Computational biology and bioinformatics, Tumour immunology, Data integration

## Abstract

Determining the diverse cell types in the tumor microenvironment (TME) and their organization into cellular communities, is critical for understanding the biological heterogeneity and therapy of cancer. Here, we deeply immunophenotype the colorectal cancer (CRC) by integrative analysis of large-scale bulk and single cell transcriptome of 2350 patients and 53,137 cells. A rich landscape of 42 cellular states and 7 ecosystems in TMEs is uncovered and extend the previous immune classifications of CRC. Functional pathways and potential transcriptional regulators analysis of cellular states and ecosystems reveal cancer hallmark-related pathways and several critical transcription factors in CRC. High-resolution characterization of the TMEs, we discover the potential utility of cellular states (i.e., Monocytes/Macrophages and CD8 T cell) and ecosystems for prognosis and clinical therapy selection of CRC. Together, our results expand our understanding of cellular organization in TMEs of CRC, with potential implications for the development of biomarkers and precision therapies.

## Abstract (web summary)

An extensive analysis of public bulk and single-cell data in colorectal cancer (CRC) patients extends immune classifications in CRC and uncovers more detail in the tumor microenvironment.

## Introduction

Colorectal cancer (CRC) remains the third most common cancer with poor overall survival and prognosis^1^. Surgery, chemotherapy and radiotherapy were commonly used treatments for CRC patients. However, the overall survival of patients is still low and it is essential to identify novel biomarkers for CRC. In addition, patients of CRC exhibit striking clinical and biological heterogeneity^2^. Besides the genetic backgrounds of CRC patients, the tumor microenvironment (TME) has been demonstrated to play important roles in the observed clinical heterogeneity and the response to cancer therapy^3^. Nevertheless, a comprehensive understanding of the TME of CRC still remains challenging.

Previous efforts have been exploring the TME of CRC and identified novel treatments. Immune checkpoint inhibitors (ICIs), in particular anti-PD-1 and anti-CTLA4 therapies, have dramatically reshaped the cancer therapy in recent years^4^. However, it has been shown that the microsatellite instable (MSI) CRC patients but not the microsatellite stable (MSS) patients respond well to the ICIs^5^. Cancer transcriptome analyses have revealed distinct immune subtypes, which spans across conventional cancer classification based on anatomical site of original^6–8^. Immune classifications of CRC facilitated the prediction of prognosis and drug response. However, these studies usually oversimplify the cell types and cellular states in TME and practical consideration have limited to limited cancer patients^9^.

Recent developments of single-cell RNA sequencing (scRNA-seq) have enabled detailed surveys of TMEs of various tumor types^10^. A single-cell analysis has informed the underlying mechanisms of myeloid-targeted therapies in CRC^11^. scRNA-seq has revealed the suppressive TME in CRC^12^ and also revealed distinct cellular factors for response to immunotherapy targeting CD73 and PD-1 in CRC^13^. Two distinct subtypes of cancer-associated fibroblasts (CAFs) were identified in CRC and demonstrated that scRNA-seq provides a unique opportunity to characterize aberrant cell states within a tumor^14^. Although such studies have provided critical insights into the TMEs of CRC, scRNA-seq studies of CRC have thus far been of moderate size. In contrast, large-scale bulk transcriptomes of CRC patients were not fully used to analysis of the TMEs of CRC.

Here, we deeply immunophenotyped the CRC by collecting the bulk and single cell transcriptome of 2350 patients and 53,137 cells. We uncovered a rich landscape of cellular states and ecosystems in CRC TMEs, including 42 cellular states and 7 ecosystems, which extended the previous immune classifications. We also validated these results across multiple independent validation CRC cohorts. Functional enrichment analysis of marker genes in cellular states revealed that the marker genes were enriched in cancer hallmark-related and immune pathways, revealing a rich functional landscape of cellular states in CRC. In addition, we revealed the prognostic atlas of cellular states and ecotypes by survival analysis. We found the cellular states and multicellular communities were significantly associated with prognosis. In particular, we identified transcriptional regulators and uncovered several possible biomarkers in CRC such as MAFK and FOXM1. Finally, we explored the potential application of cellular states and ecotypes in treatment selection and found that 12 cellular states and ecotypes CE7 were with the potential to predict therapeutic response. Taken together, our large-scale transcriptome integrative analysis provides a comprehensive atlas of clinical associated TMEs in CRC.

## Results

### Overview of cell states and ecosystem profiling in CRC

**Fig. 1:**
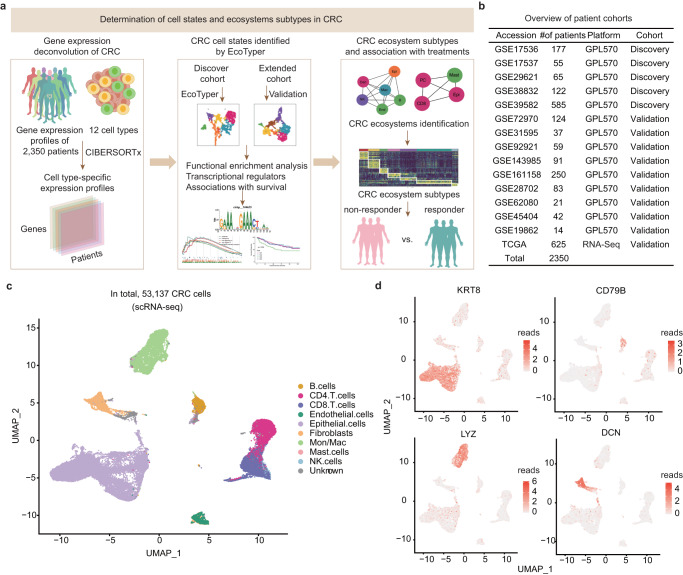
Framework for high-throughput characterization of cellular states and ecosystems in CRC. **a** Overview of the application of EcoTyper to cellular states and ecosystems profiling in CRC. **b** Summary of CRC patient cohorts of bulk transcriptomes. **c** UMAP visualization of scRNA-seq transcriptome of CRC patients. **d** UMAP visualization of expression of several marker genes.

We employed EcoTyper to illuminate cell states and ecosystems from bulk and single-cell transcriptome of CRC patients (Fig. 1a and Supplementary Fig. 1). The first step of EcoTyper was to apply CIBERTSORTx to impute cell type specific gene expression profiles and provided an estimation of the abundance of cell types in a mixed cell population. Transcriptionally defined cell states in each cell type were identified in discovery CRC cohorts and cell state co-occurrence models that defined cell communities were identified. The identified cell states in CRC were recovered separately in independent validation CRC cohorts, including single-cell data (Fig. 1a). The clinical associations of cell states and ecotypes with patient survival (including overall survival and progression-free survival) were analyzed in CRC. Moreover, we also explored the potential utility of cell states and ecotypes for clinical therapy of CRC patients (Fig. 1a and Supplementary Fig. 1).

To realize cell states and multicellular community ecotypes of CRC, we collected 1725 CRC samples of 14 GEO datasets and 625 CRC samples from TCGA project (Fig. 1b, Supplementary Fig. 1 and Supplementary Data 1). The integrated gene expression profiles of 1004 CRC samples from five GEO datasets were used as discovery cohorts and the remaining 10 GEO or TCGA datasets were used as independent validation cohorts, respectively. To further validate the predefined cell states and multicellular communities at the single-cell level, we collected two single-cell transcriptome of CRC from recent studies^15,16^. In total, 53,137 high quality cells were annotated to 9 cell types (Fig. 1c), which were the major components in EcoTyper. In addition, we found that there was no study-based bias in the integrated single cell transcriptome (Supplementary Fig. 2). Particularly, we found that several representative marker genes exhibited high expression in corresponding cell types (Fig. 1d). The epithelial marker KRT8 highly expressed in epithelial cells, CD79B was highly expressed in B cells, and DCN was highly expressed in fibroblast cells.

### The landscape of cellular states in TME of CRC

**Fig. 2:**
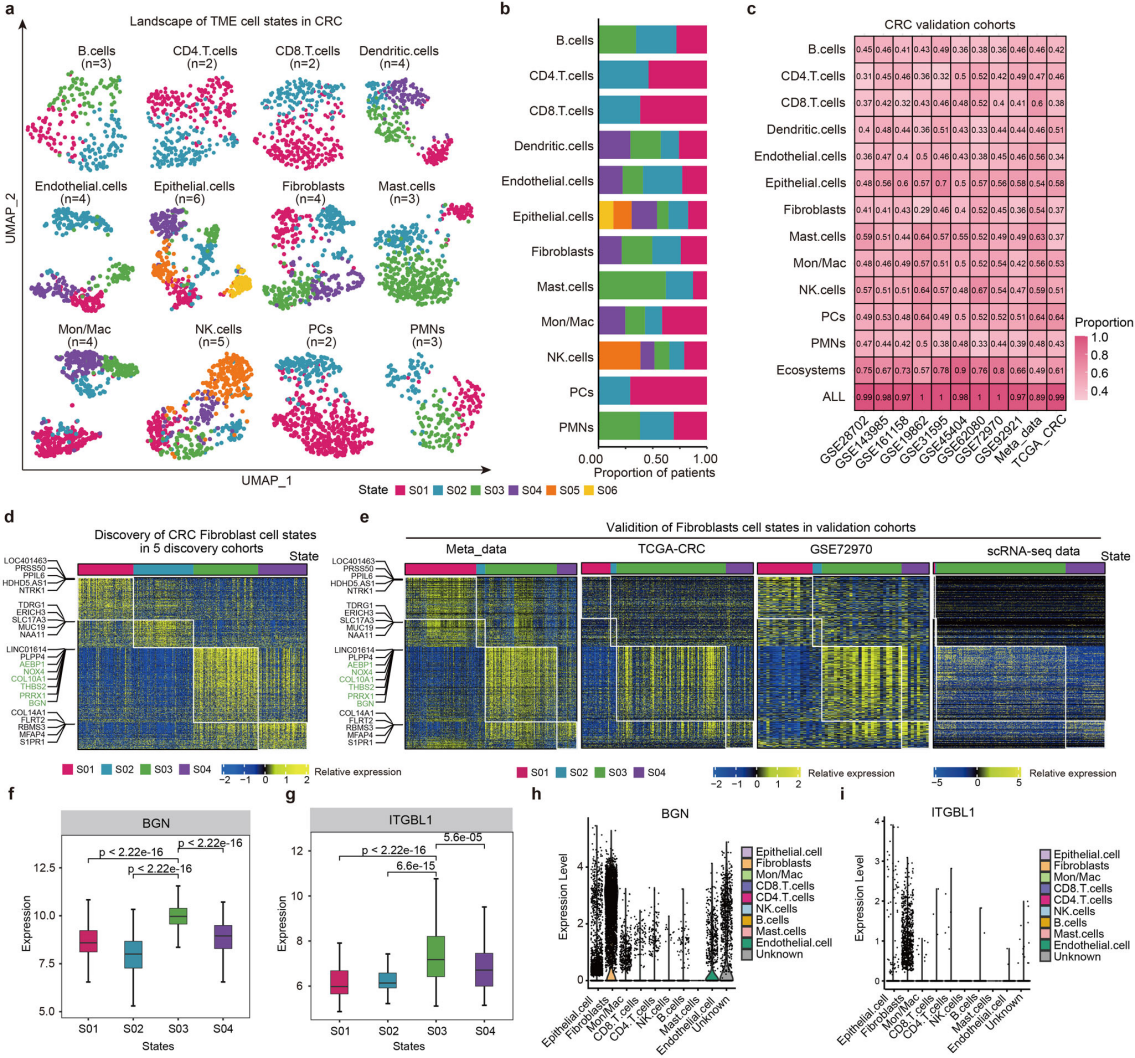
The cellular states landscape of CRC. **a** UMAP projection of cellular states across CRC tumor patients in the discovery cohort. Points are colored by the most abundant cellular state per sample, and the colors are for S01-S06 states. **b** The proportions of patients within different cellular states in each cell type. **c** Heatmap showing the proportion of CRC patients recovered in validation cohorts. ALL is for union of all cellular states. **d** Heatmap depicting four fibroblast cell states identified from CRC bulk transcriptomes of discovery cohorts. Patient samples are organized by the most prevalent cell states and genes used for discovery of the cell states are shown. **e** Heatmap depicting four fibroblast cell states in validation cohorts and single cell transcriptome. The color gradients in Fig. 2d, e showing relative expression, which is the log2 fold change in one state to the others. **f**, **g** Boxplot showing the expressions of BGN and ITGBL1 in patients within four cell states. **f** for BGN and **g** for ITGBL1. The lines in each box plot represent median values, and the box limits represent upper and lower quantiles. **h**, **i** Violin plots showing the expressions of BGN and ITGB1 across cell types in single cell transcriptome. **h** for BGN and **i** for ITGBL1.

We next decoded the cellular heterogeneity across the assembled 1004 CRC patients in the discovery cohort. Cell type abundance was estimated to yield 12 cell type-specific gene expression profiles by CIBERSORTx. Based on the EcoTyper framework, we obtained 42 distinct cellular states from 12 cell types, ranging from 2 to 6 states per cell type (Fig. 2a). The proportions of patients in each cell type were greatly different (Fig. 2b). The majority of patients were within mast cell state S03 whereas were within PCs cell state S01. To corroborate the cellular states in CRC, the identified cell states were recovered using 11 independent validation cohorts, and the accuracy was evaluated according to the proportion of samples of different cell types. We found that the cell states were recovered in 89% to 100% patients in the validation cohorts (Fig. 2c) and the majority of cell states were significant recovered (Supplementary Data 1 and 2), suggesting the cell states in CRC were robust.

To characterize the cellular states in CRC, we started with the fibroblast cells. Cancer-associated fibroblasts are key components of the tumor microenvironment and can promote or inhibit tumor growth^17,18^, playing an important role in CRC^19^. Although the origin of cancer-associated fibroblasts in CRC is unclear, there is growing evidence that fibroblasts are a major source^20^. We obtained four cellular states in fibroblasts (Fig. 2a, d), for each independent validation cohort, we were able to reproduce the cellular states in Meta_data, TCGA, GSE72970 and scRNA-seq cohorts (Fig. 2e). We also found that several genes associated with development and progression in CRC were significantly highly expressed in S03, including AEBP1, NOX4, COL10A1, THBS2, PRRX1, ITGBL1 and BGN. AEBP1 is a marker of cancer-associated fibroblasts, and high expression of COL10A1 was associated with unfavorable prognosis in CRC^21^. In particular, BGN and ITGBL1 were highly expressed in S03 in bulk and single cell datasets (Fig. 2f–i). It has been demonstrated that BGN was fibroblast-specific biomarker of poor prognosis in CRC^18^. Moreover, we also found that BGN and ITGBL1 were highly expressed in metastatic CRC than primary patients (Supplementary Fig. 3a, b).

In addition, tumor-associated macrophages are the major player in tumor microenvironment that frequently associated with tumor metastasis^22^. We identified four Monocytes/Macrophages cell states in CRC (Supplementary Fig. 3c) and the results in the discovery cohorts were validated in bulk validation cohorts and scRNA-seq data (Supplementary Fig. 3d). There were several genes highly expressed in each state. For example, PPP3CB and UBE3B were highly expressed in S01, whereas ECM2, THBS2, CYP1B1, MGP, ANTXR1, CD14 showed higher expression in S04 (Supplementary Fig. 3e). Thrombospondin 2 (THBS2), as a secreted protein, was confirmed to be highly expressed in different cancers including colorectal cancer and its high expression was associated with poor prognosis^23,24^. We also obtained the similar results for fibroblasts and Mon/Mac cell states in another three bulk and one single cell data (Supplementary Fig. 4), as well as the combined validation bulk and single-cell CRC transcriptomes (Supplementary Fig. 5 and Supplementary Fig. 6). Together, these results suggest that integrative analysis of transcriptome we derived a high-resolution cell state atlas of 12 cell types in CRC.

### Comprehensive functional profiling of cell states in CRC

**Fig. 3:**
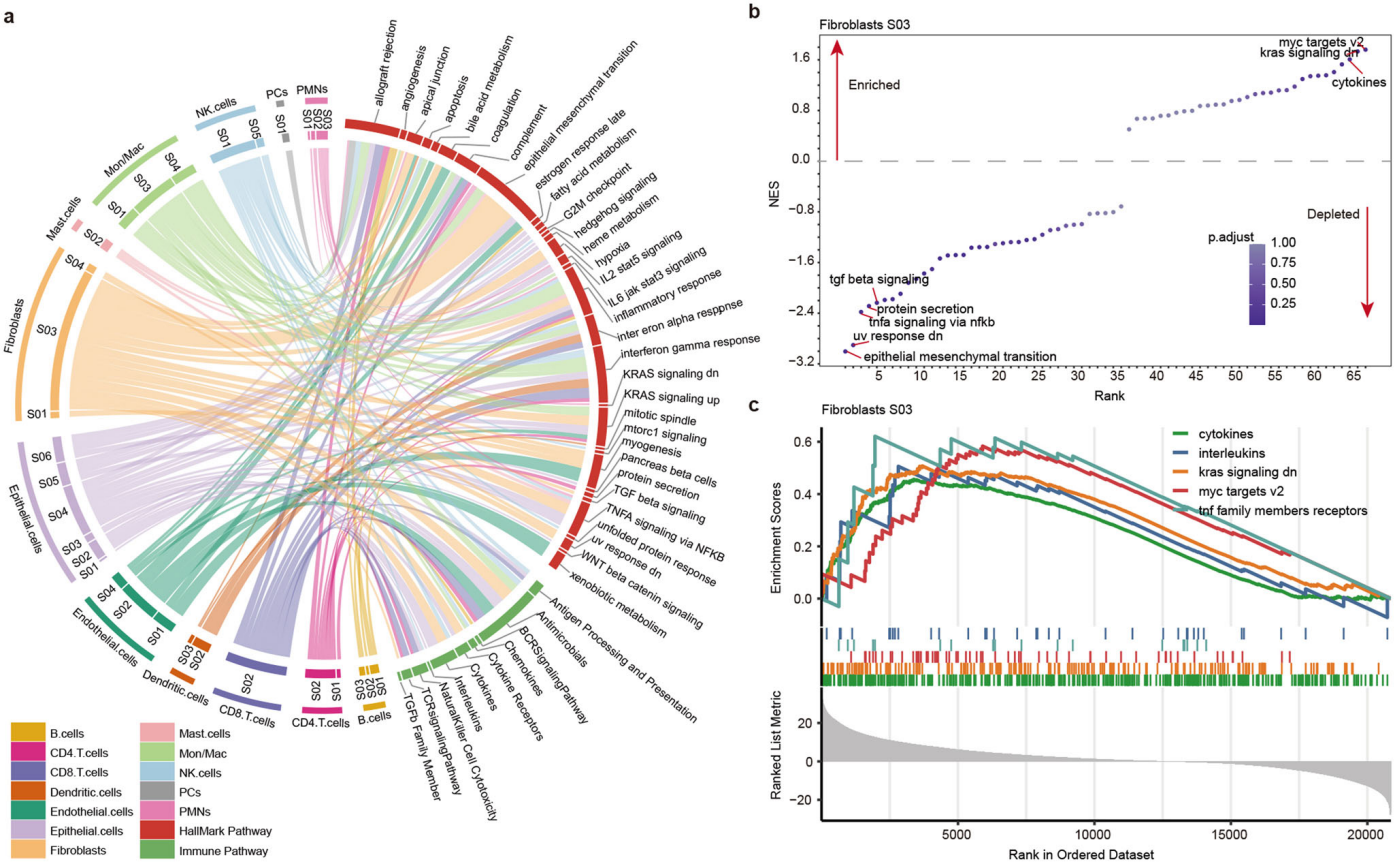
Comprehensive functional profiling of cellular states in CRC. **a** Circos plot depicting the enriched cancer hallmark and immune-related pathways for genes highly expressed in cellular states in CRC. Cellular states across cell types are organized on the left and pathways are on the right, widths of the links are corresponding to the proportion of genes enriched in pathways. **b** Scatter plot showing the normalized enrichment scores (NES) of pathways in fibroblasts S03 cell states. Pathways are ranked by NES. Colors of dots are corresponding to the *p*-values. **c** The enrichment score (ES) distribution for the genes differentially expressed in fibroblast cell state S03 enriched in five pathways. Each line is for a pathway.

Having identified the cellular states for 12 cell types in CRC, we next investigated the potential functions of each cell state. Based on the marker genes in cellular states, we first performed functional enrichment analysis and found that the majority of marker genes were enriched in cancer hallmark-related and immune pathways (Fig. 3a and Supplementary Data 3). For example, we found that numerous cancer hallmark-related pathways were significantly enriched in fibroblasts and epithelial cellular states, such as epithelial mesenchymal transition (EMT), TGF beta signaling and TNFA signaling via NFKB (Fig. 3a). It has been demonstrated that the interaction between cancer cells and the surrounding cancer‑associated fibroblasts (CAFs) can markedly affect the tumor cells growth, metabolism, metastasis, and progression^25^. We next analyzed the biological processes in GO and found that the majority of the marker genes of cellular states were significantly enriched in a number of regulation and response to extracellular stimulus pathways (Supplementary Fig. 7a).

We next investigated the S03 state of fibroblasts in detail and found that three cancer hallmark-related pathways were significantly enriched by marker genes, including MYC targets, KRAS signaling down and cytokines (Fig. 3b, c). In contrast, several oncogenic pathways (i.e., TGFβ signaling, EMT and UV response) were depleted in this cellular state. These results suggested that S03 cellular state of fibroblasts might play a tumor suppressor role in CRC. In addition, we found that genes highly expressed in S02 state of CD4 T cells were significantly enriched in inflammatory response, chemokine receptor, cytokines and chemokine pathways, but depleted in G2M checkpoint, E2F targets and MYC targets (Supplementary Fig. 7b and Fig. 7c). Detail analysis of the marker genes we found that patients in S02 state of CD4 T cells exhibited high expression of CD8A, LCP2 and CD48. CD8A has been found to highly express in low-risk CRC patients that are response to pembrolizumab treatment^26^. LCP2 is involved in T cell activation and can increase the IL-2 gene promoter activity following transient overexpression, which is associated with better prognosis of cancer patients^27^. Together, these results uncovered a rich functional landscape of the cellular states in CRC.

### Deciphering the prognostic atlas of cell states in CRC

**Fig. 4:**
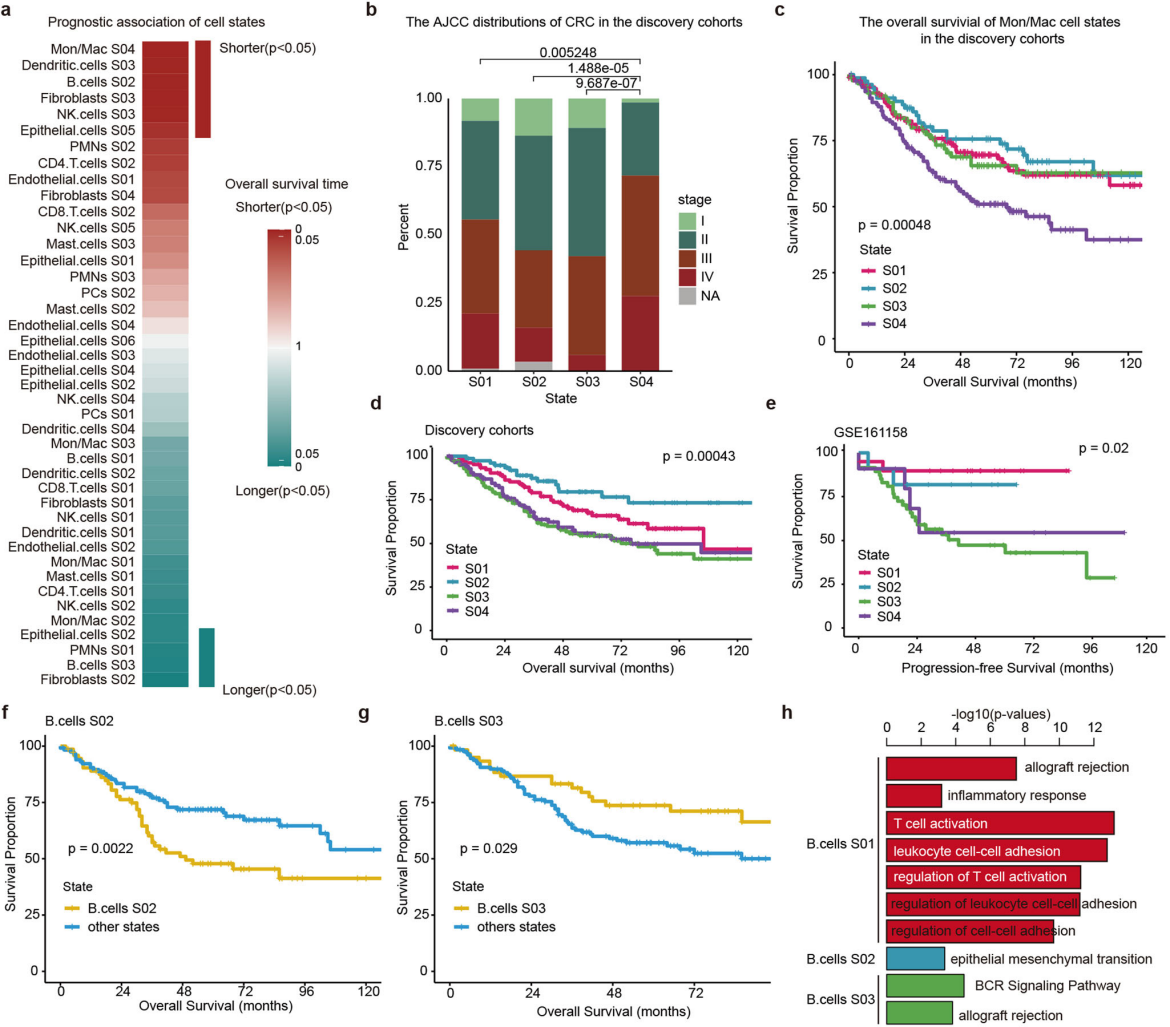
Cell-state-specific survival associations in CRC. **a** Cellular state-specific survival associations in the CRC discovery cohorts. Heatmap showing the *p*-values, and red for the risky and cyan for protective cellular states. **b** Barplots showing the proportion of patients in different stages across four Mon/Mac cellular states. **c** Kaplan–Meier plots showing differences in overall survival between patients within four Mon/Mac cellular states. **d** Kaplan–Meier plots showing differences in overall survival between patients within four fibroblast cellular states in discovery cohorts. **e** Kaplan–Meier plots showing differences in progression-free survival between patients within four fibroblast cellular states in validation cohorts. **f**, **g** Kaplan–Meier plots showing differences in overall survival between patients within B cell S02, B cell S03 and other states. **f** for B cell S02 and **g** for B cell S03. **h** Barplots showing the enriched functional pathways for genes highly expressed in three B cell states.

It has been shown that the cell-states can predict cancer clinical outcomes^9,28^. However, their relationships with prognosis have not been thoroughly analyzed in CRC. We therefore charted the prognostic atlas of cell states in CRC. Global survival associations dichotomized all evaluated cell sates into favorable and adverse states (Fig. 4a and Supplementary Data 4). Six cell states, including S04 of Monocytes/Macrophages, S04 and S03 of dendritic cells were found to be associated with adverse survival (Supplementary Fig. 8a–e) and four cell states (S02 of fibroblast, S03 of B cells, S02 of Epithelial cells and S01 of PMNs) were significantly associated with favorable survival (Fig. 4a and Supplementary Fig. 8f, g). In particular, we analyzed the four cell states of Monocytes/Macrophages in detail. We found that the patients within four cell states have significantly different AJCC stages and more patients of cell state S04 were in III and IV stages (Fig. 4b). Significant differences in survival were found among the patients within four states (*p* = 0.00048, log-rank test), and cell state S04 was associated with adverse survival of CRC patients (Fig. 4c). Functional analysis of the marker genes in S04 revealed numerous cancer-related pathways, such as epithelial mesenchymal transition and inflammatory response pathways (Supplementary Data 3).

We next analyzed the association between cell states and survival in fibroblasts, and found that patients in four states have significant differences in survival in the discovery cohorts (Fig. 4d, *p* = 0.00043, log-rank test) and validation cohorts (Fig. 4e, *p* = 0.02, log-rank test), and cell state S03 was associated with poor survival in CRC. These results were consistent with the observations that the marker genes in S03 were significantly enriched in a large number of cancer pathways, such as epithelial mesenchymal transition, TNFA signaling via NFKB and hypoxia (Supplementary Fig. 8h). Moreover, the global association analysis also revealed the biological and clinical heterogeneity of cell types. For example, B cell types annotated as S02 and S03 were associated with shorter and longer survival time, respectively (Fig. 4f, g, *p* = 0.0022 and 0.029, log-rank tests). Functional analysis revealed that the patients within S02 highly expressed genes in epithelial mesenchymal transition, whereas patients in S03 highly expressed genes in B cell receptor signaling pathway (Fig. 4h). These results were consistent with the associations with patient survival.

### Characterization of multicellular ecosystems in CRC

**Fig. 5:**
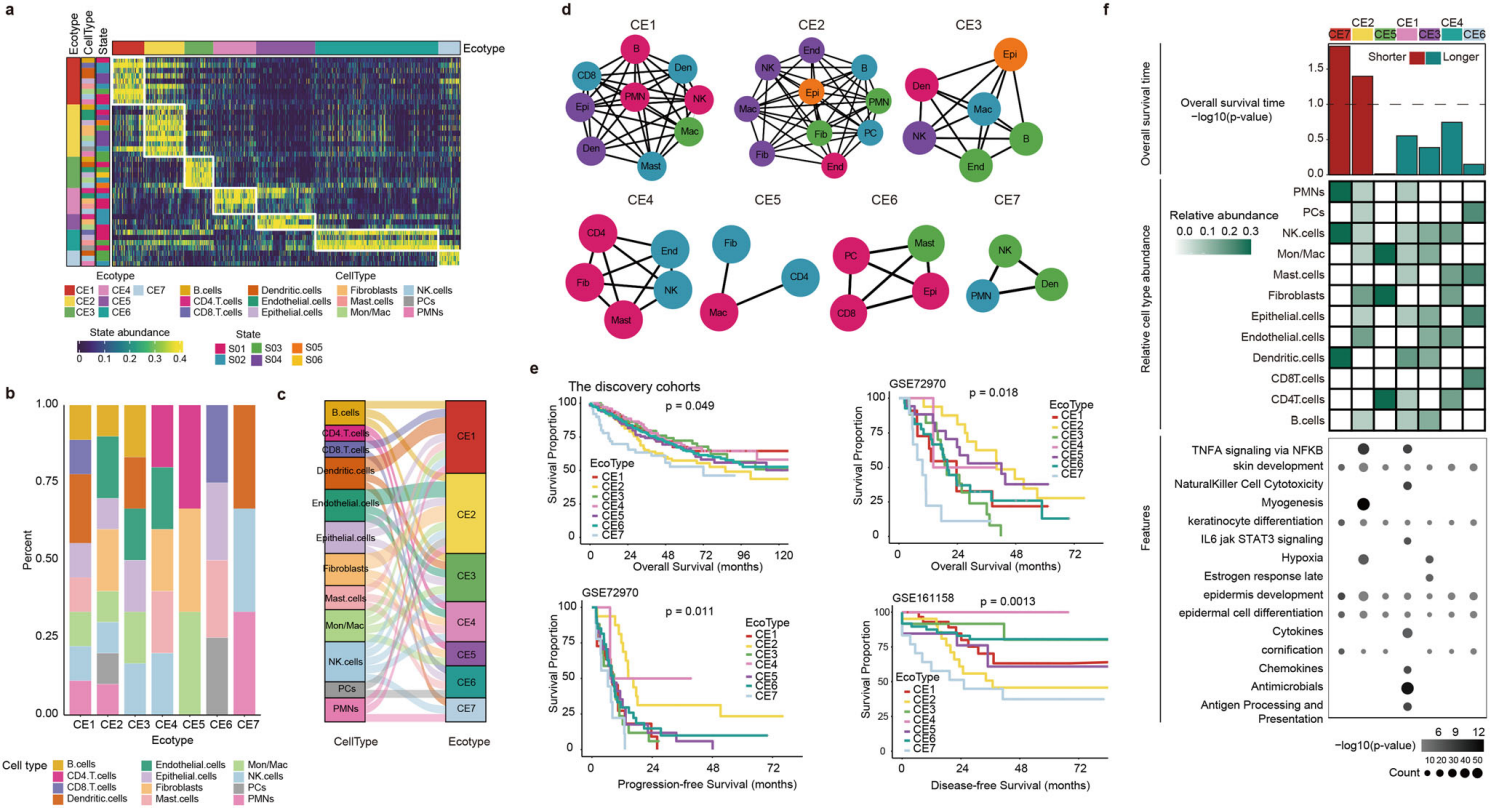
Landscape of cellular ecosystems in CRC. **a** Cell state abundance patterns in the CRC discovery cohort, with cell states organized into seven colorectal ecotypes (CEs) and tumor samples (columns) ordered by the most abundant CE per sample. **b** The proportions of cell types in seven CEs in CRC. **c** Distribution of cellular states in seven ecotypes. **d** Network organization of cellular states in seven ecotypes. **e** Kaplan–Meier plots showing differences in survival between patients within seven CEs, including the overall survival in discovery and validation cohorts, progression-free and disease-free survival in the validation cohorts. **f** Molecular characteristics of seven ecotypes. The relationship between seven ecotypes and prognosis, dotted line expression *p* = 0.05 (top), the abundance of cell types in seven ecotypes (middle), the functional pathways of seven ecotypes (bottom).

Tumors are comprised by complex ecosystems and decoding the ecosystems is important to understand the mechanisms of cancer development and progression. We next applied EcoTyper to discover the multicellular community ecotype in CRC. In total, seven multicellular communities, which were termed as colorectal ecotypes (CEs) were identified in CRC (Fig. 5a and Supplementary Data 5). We next aggregated the cell-state abundance profiles and assessed the CE composition in a continuous manner. We found that nearly every CRC patient had a dominant CE, while numerous tumors were comprised of multiple CEs (Fig. 5a). Seven CEs were reproducible in independent validation cohorts (Supplementary Fig. 9), and the recovery ratio of patients exceeded 90% (Fig. 2c), underscoring the stability of identified ecotypes in CRC. We found the CEs varied substantially in the constituent cell types and cellular states (Fig. 5b, c). The variety of cell types were the largest in CE1 and CE2, which were constituted by 8 cell types (Fig. 5b), whereas CE5 and CE7 contained only three cell types. Moreover, we found that CEs contained 3 to 10 cellular states and the compositions of cellular states were varied greatly among CEs (Fig. 5d).

Since the CE profiling has been demonstrated to improve clinical outcome prediction^9^, we next analyzed the relationship between CEs and CRC survival. We found that there were significant differences in survival of patients within CEs (Fig. 5e, *p* = 0.049 in discovery cohort, *p* = 0.018 for overall survival, *p* = 0.011 for progression-free survival and p = 0.0013 for disease-free survival in validation cohort, log-rank tests). In addition, CRC patients in CE7 and CE2 have poor prognosis (Fig. 5e). To understand the functions of CEs in CRC, we performed functional enrichment analysis. We found that genes highly expressed in CE7 were enriched in development and differentiation-related pathways, whereas genes highly expressed in CE2 were enriched in TNFA signaling via NFKB, myogenesis and hypoxia signaling pathways (Fig. 5f). These results suggest that multicellular communities can capture biological pathways with predictive value in clinical outcome.

### Transcriptional regulators of TME in CRC

**Fig. 6:**
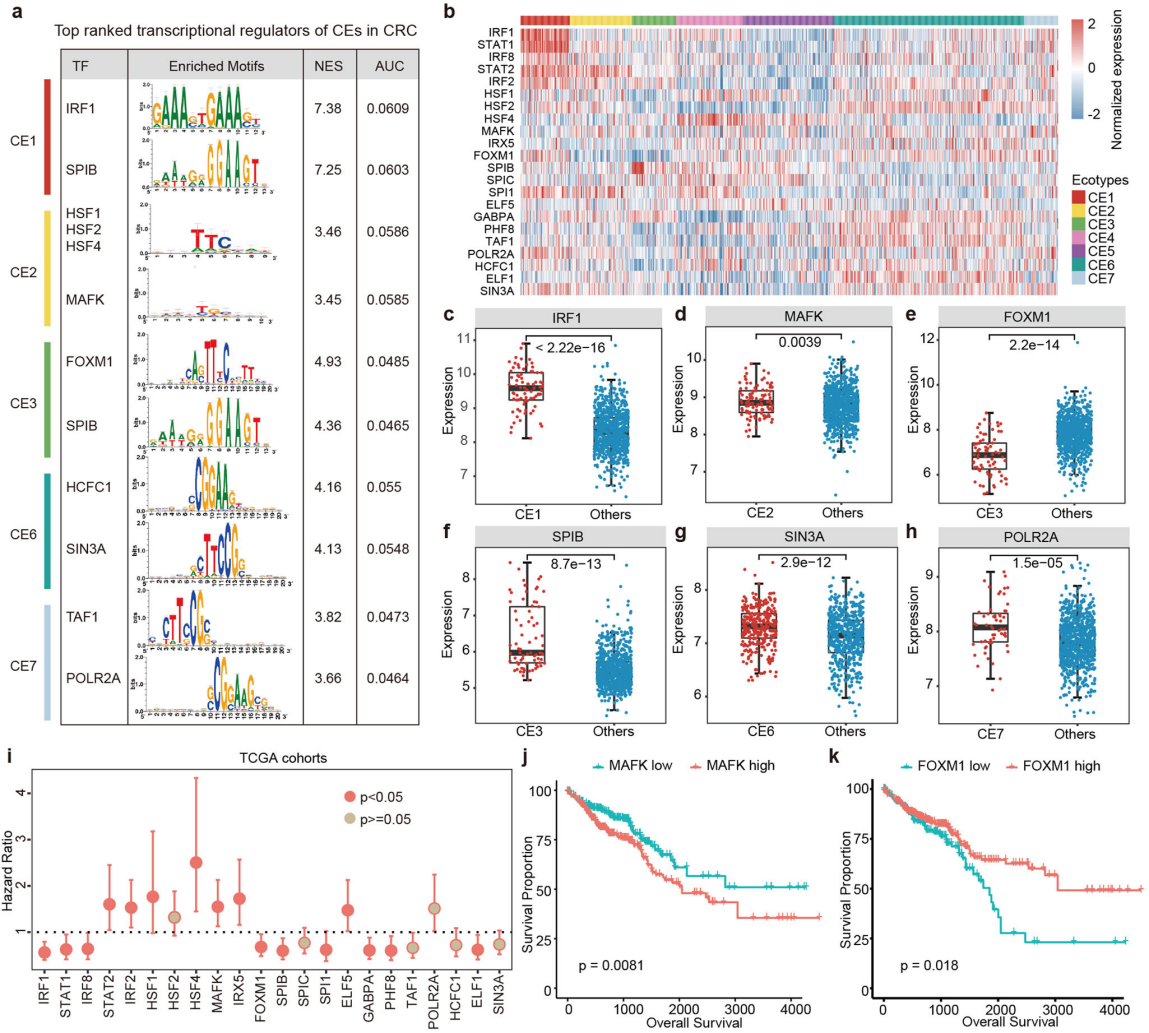
Transcriptional regulators of cellular ecosystems in CRC. **a** Representative TFs potentially regulating the genes highly expressed in corresponding CEs. Top two TFs were shown for each CE in CRC. **b** Heatmap depicting the expressions of TFs across patients within different CEs. **c**–**h** Boxplots showing the expressions of representative TFs. **c** for IRF1, **d** for MAFK, **e** for FOXM1, **f** for SPIB, **g** for SIN3A and **h** for POLR2A. Median and quartiles were the median and quantiles expression values of genes. **i** The hazard ratios (HRs) and 95% confidence levels for the expressions of TFs associated with clinical survival. Centre point is the HR, and bounds are the 95% confidence levels. **j** Kaplan–Meier plots showing differences in survival between patients with high and low expression of MAFK. **k** Kaplan–Meier plots showing differences in survival between patients with high and low expression of FOXM1.

To further delineate the functions of CEs, we next explored the upstream transcriptional regulators in CRC. Integrating the marker genes in CEs and the transcription factors (TFs) binding sites, we identified the TF regulators enriched in five CEs in CRC (Fig. 6a and Supplementary Fig. 10a–e). IRF1 is a member of the IRF family and has been involved in the development of multiple tumors. We found that the target genes of IRF1 were significantly enriched in genes that highly expressed in CE1 (Fig. 6a). It has been shown that IRF1 regulates the progression of CRC via interferon‑induced proteins^29^. FOXM1 has been involved in malignant behaviors of cancer and was demonstrated to trigger aggressiveness of CRC^30^. We found that the genes highly expressed in CE3 were likely to be targets of FOXM1 (Fig. 6a). In addition, POLR2A has been identified to be always co-deleted with TP53 in human cancers, and suppression of POLR2A can inhibit the proliferation, survival and tumorigenic potential of CRC cells^31^. We found that POLR2A can potentially target the genes highly expressed in CE7 (Fig. 6a), which might explain the observations that patients in CE7 were with poor survival (Fig. 5f).

We next investigated the expression of top five ranked TFs enriched in each CE. We found that TFs exhibited significantly higher expression in corresponding CEs (Fig. 6b–h and Supplementary Fig. 10f–i). For example, IRF1 exhibited significantly higher expressions in CE1 (Fig. 6c) and FOXM1 and SPIB exhibited significantly lower and higher expressions in CE3 (Fig. 6e, f). SIN3A and PHF8 were highly expressed in patients within CE6 (Fig. 6g and Supplementary Fig. 10i), and POLR2A exhibited significantly higher expression in patients within CE7 (Fig. 6h). Moreover, we investigated the associations between TF expression and clinical outcome. We found that the expressions of 63.64% and 72.73% of top ranked TFs were associated with patient survival in the discovery and TCGA CRC cohorts (Fig. 6i and Supplementary Fig. 10j). High expression of MAFK was associated with poor survival of CRC patients (Fig. 6j, log-rank *p* = 0.0081) and high expressions of FOXM1 and IRF1 were associated with better survival of patients (Fig. 6k and Supplementary Fig. 10k, *p*-values = 0.018 and 0.00032). Collectively, these data reveal extensive TF regulators that play important roles in the TME of CRC.

### Cellular states and CEs predict therapeutic benefits in CRC

**Fig. 7:**
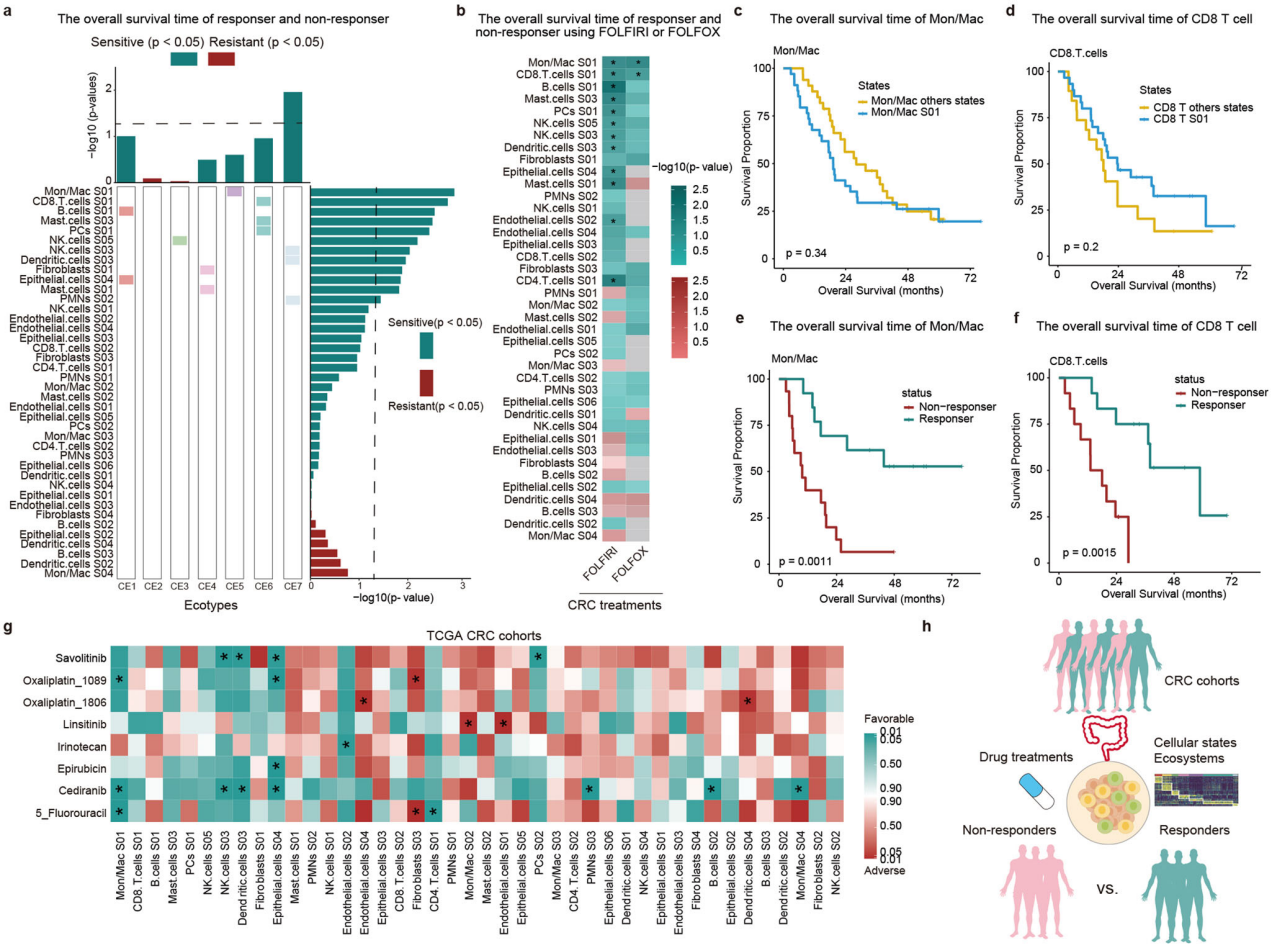
Prediction of response to drug treatments in CRC based on cellular states. **a** Association between cellular states and therapeutic benefit from FOLFIRI and FOLFOX treatments in CRC. Cell states were ranked by *p*-values of the associations with overall survival. **b** The association between cellular states with therapeutic benefits for FOLFIRI and FOLFOX treatment, separately. **c** Kaplan–Meier plots showing differences in survival between patients with Mon/Mac S01 and other states. **d** Kaplan–Meier plots showing differences in survival between patients with CD8 T cells S01 and other states. **e** Kaplan–Meier plots showing differences in survival between responders and non-responders with Mon/Mac S01 states. **f** Kaplan–Meier plots showing differences in survival between responders and non-responders with CD8 T cells S01 states. **g** Heatmap showing the association between cellular states and therapeutic benefits for drugs used for CRC therapy. TCGA CRC cohorts were used. **h** Schematic diagram showing the prediction of response to drug treatments by considering the cellular states in CRC. *p < 0.05.

Next, we asked whether the cellular states or CEs can predict therapy response of CRC. We predicted the therapeutic benefit by comparing the survival times of responders and non-responders, and found that 12 cellular states in which the overall survival of responders was significantly better than that of non-responders (Fig. 7a). In addition, the progression-free survival of responders was significantly better than that of non-responders in 9 cellular states (Supplementary Fig. 11a). These results suggested that fluorouracil-based adjuvant chemotherapy (ACT) had a better therapeutic benefit in these cellular states. Monocytes/Macrophages S01 and CD8 T cell S01 were the most significantly associated with therapeutic benefit of CRC (Fig. 7a, *p*-values = 0.0011 and 0.0015), indicating that fluorouracil-based ACT might be most suitable for CRC patients within two cellular states. Also, for ecotypes, we found the treatment was beneficial to patients with CE7, which included three cell states (NK cells S03, Dendritic cells S03 and PMNs S02) that were sensitive to drug treatment of CRC (Fig. 7a). We next compared the benefits of FOLFOX and FOFIRI therapies in each cellular state respectively. We found that Monocytes/Macrophages S01 and CD8 T cell S01 were effective to both two treatments (Fig. 7b). In contrast to FOLFOX, there were more cellular states response to FOLFIRI treatment (Fig. 7b), which were consistent with the observations in combination analysis (Fig. 7a). We next investigated the Monocytes/Macrophages S01 and CD8 T cell S01 cellular states in detail. We found that patients harboring high levels of Monocytes/Macrophages S01 and CD8 T cell S01 exhibited no differences in survival when compared with patients of other Monocytes/Macrophages and CD8 cellular states (Fig. 7c, d, *p* = 0.34 and 0.2, log-rank tests). Furthermore, when patients were stratified by treatment response status, we found that the patients that were response to treatments had better survival than the non-responders in CRC patients harboring high levels of Monocytes/Macrophages S01 and CD8 T cell S01 levels (Fig. 7e, f, *p* = 0.0011 and 0.0015, log-rank tests).

To determine whether the cellular states were associated with additional cancer therapy, we extended our analysis to 198 drug treatments for CRC patients. We found that the cellular states can potentially predict therapeutic benefits for a large number of drugs in CRC (Supplementary Data 6). There were more drug treatments can benefit from the dendritic cells S03 and epithelial cells S04 cellular states (Supplementary Data 6). In particular, we investigated five drugs commonly used in treatments of CRC (Fig. 7g). We found that patients resistant to 5-Fluorouracil, cediranib and oxaliplatin treatments were with worse survival within Mon/Mac S01 cellular state (Supplementary Fig. 11b–d, *p* = 0.015, 0.018 and 0.038, log-rank tests). Together, these data suggest that understanding the cellular states and ecotypes can capture biological signals with predictive values in drug treatment response (Fig. 7h).

## Discussion

Emerging studies have shown that the diversity of TME plays an important role in the occurrence, development and metastasis of CRC. It has prompted researchers to stratify patients by transcriptome-defined subtypes based on cell type-specific gene expression patterns in cancer. High-resolution characterizations of the TMEs have been performed in various cancer types, such as diffuse large B cell lymphoma^28^ and pan-cancer study^9^. In this study, we employed EcoTyper to characterize the TMEs of large-scale CRC patients. In total, 42 transcriptome-defined cellular states were identified, which further clustered into seven multicellular communities. In addition, we investigated whether the cell states identified in discovery cohorts could be recovered in validation cohorts by EcoTyper. We found that the majority cell states can be significantly recovered (Supplementary Data 2). In addition, there were numerous methods for mapping the probe to gene and metrics to assign similar samples^32–34^. To validate the robustness of the cellular states in CRC, for multiple probes mapping to the same gene we took the maximum value, we found that the majority of cellular states identified in discovery cohorts can be significantly recovered in this validation cohort (Supplementary Fig. 12). These results suggested that the noted cellular states are not due to technology differences. This systems-level landscape of cellular states and ecotypes provides insights into the CRC heterogeneity.

The results of this work have important implications for clinical treatment of CRC. Our analysis identified the cellular states of CRC composed of hundreds of genes, which are expected to contribute to the malignant behaviors of tumors and play important roles in TMEs. Functional enrichment analysis revealed that genes highly expressed in cellular states were significantly enriched in cancer hallmarks and immune-related pathways. In addition, we analyzed the cell state composition of these 12 cell types and their relationships with survival. Six cell states were significantly associated with favorable prognosis, while four cell states were associated with adverse prognosis. BGN, ITGBL1, WNT2, and IL6 were significantly expressed in fibroblast cellular state S03, suggesting their important roles in CRC. Our recent studies have demonstrated that TMEs have been strictly regulated by a series of regulators^35–37^. To systematically identify the transcriptional regulators for cellular states, we performed the TF binding sites enrichment analysis and identified a number of critical TFs in CRC, such as IRF1, MAFK, FOXM1 and POLR2A. These TFs are valuable candidates for functional validation in cell lines or animal models.

Heterogeneity in CRC may also determine the sensitivity of clinical chemotherapy. We stratified CRC patients by cellular states and ecotypes to predict potential therapeutic benefits. We found that in 12 cellular states, including Monocytes/Macrophages S01 and CD8 T cells S01, drug treatments improved patient survival, but FOFIRI or FOLFOX had no significant effect on patients within other cell states. Moreover, we predicted the responses of 198 drug treatments and provided the landscape of cellular states to therapy in CRC. We also found that patients resistant to 5-Fluorouracil, cediranib and oxaliplatin treatments were with worse survival within Monocytes/Macrophages S01 cellular state. As the prediction is based on cellular states and ecotypes, we think the rich bulk RNA-seq analysis would suffice to predict therapeutic benefits. Although predictions of therapeutic benefits based on cellular state and ecotype will need to be validated with more data, our results provided opportunities for diagnostic and therapeutic strategies by considering the tumor cellular states and ecosystems.

In summary, we deeply immunophenotyped the TMEs of CRC patients by comprehensive analyses of large-scale bulk and single cell transcriptomes. Our results provide directions for predicting prognosis and potential therapeutic benefits, thereby facilitating better treatment options in cancer.

## Methods

### Collection of public bulk CRC transcriptome

In total, genome-wide gene expression profiles of 1725 CRC patients from 14 independent public datasets were collected from Gene Expression Omnibus (GEO) and The Cancer Genome Atlas (TCGA) (Supplementary Data 1). The datasets from GEO were retrieved from the Affymetrix® GPL570 platform (Human Genome U133 Plus 2.0 Array). We downloaded the raw CEL files of 14 GEO datasets, and processed via the robust multiarray averaging (RMA) algorithm implemented in the ‘affy’ package in R program^38^. The ‘sva’ R package^39^ was used to remove batch effects for the combined data. Principal components analysis (PCA) was used to detect the results by ‘prcomp’ R function. According to the annotations in ‘hgu133plus2.db’, all probes were mapped to gene symbols. We removed the probes that mapped to multiple genes and for multiple probes mapping to the same gene we took the average value. There were 20,862 genes remained for analysis in the microarray platform.

Similar as one previous study^26^, five datasets (GSE17536, GSE17537, GSE29621, GSE38832 and GSE39582), including 1004 CRC patients encompassing complete overall survival (OS) and disease free survival (DFS) information were used as discovery cohorts. To obtain the gene expression profile in discovery cohorts, we used the ‘ComBat’ function to perform batch correction^40^. The RNA-Seq based gene expression of colon adenocarcinoma (COAD) and rectum adenocarcinoma (READ) projects were integrated together and referred as TCGA-CRC, which included 625 patients. In total, ten datasets including 1346 colorectal cancer patients were used as the validation cohorts (Supplementary Data 1). Moreover, we intenerated the GEO datasets in validation cohorts as a metadata, and it was also used as a validation cohort. All relevant ethical regulations were followed the original study of the datasets and the authors of the source studies had also obtained informed consent from participants.

### scRNA-seq data of CRC patients

We collected two scRNA-seq transcriptome data of CRC patients, including GSE132465 (*n* = 23)^15^ and E-MTAB-8107 (*n* = 7)^16^. Raw count matrix for each sample was converted into a Seurat object using the R package Seurat^41^. To filter low quality cells, outlier cells were identified by: log (UMI counts) (>2 MADs, both end), log (number of genes expressed) (>2 MADs, both end) and log (percent mitochondrial read count +1) (>2 MADs, high end). Next, we normalized the scRNA-seq dataset using the ‘NormalizeData’. The function ‘FindVariableFeatures’ was used to identify the top 2000 highest variable genes with method=’vst’. We assigned per cell cycle phase by calculating cell cycle score with ‘CellCycleScoring’ followed by regressed out with ‘ScaleData’. The cycle genes were obtained from Seurat. To filter potential doublets, we used ‘scDblFinder’ and ‘DoubletFinder’. Only cells identified as doublets by both two methods were removed from our analysis.

For the transcriptome from E-MTAB-8107, we used SingleR to annotate the cell types^42^. Cell type annotations for GSE132465 were obtained from the corresponding study. After quality control and doublet removal, canonical correlation analysis (CCA) was used to integrate cells of two datasets^41^. Top 20 principal components were selected for dimensionality reduction and visualization.

### Cell type-specific gene expression purification in CRC

To determine the cell type-specific gene expression profiles in CRC patients, we used CIBERSORTx^43^ to impute gene expression profiles and provide an estimation of the abundances of various cell types. First, we used CIBERSORTx to estimate the proportions of cell type in a patient from bulk CRC transcriptome. To analyze the major cell populations in CRC, we applied the signature matrix in EcoTyper^9^, which includes 12 major cell types. This signature matrix is consisted of two previously validated signature matrixes, LM22^44^ and TR4^43^. LM22 is a signature matrix consisting of 22 human immune subsets, which were maped into 9 major lineages: B cells, plasma cells (PC), CD8 T cells, CD4 T cells, natural killer (NK) cells, Monocytes/Macrophages, dendritic cells, mast cells, polymorphonuclear neutrophils (PMNs). TR4 is a signature matrix consisting of epithelial, endothelial, immune and fibroblast populations. We estimated the abundance of 12 cell types based on the bulk transcriptome of discovery cohorts. Next, the sum of the abundance of 12 cell types in each patient was normalized to 1. Finally, we employed CIBERSORTx to determine cell type-specific gene expression profiles with default parameters.

### Discovery of CRC cell states

Once we determined the cell type-specific gene expression profiles, we used EcoTyper to identify clusters for each cell type. Based on the purified gene expression profiles, EcoTyper uses non-negative matrix factorization (NMF) combined with specific heuristics to identify and quantitate cell states^9^. The cluster number was ranged from 2 to 20 and the cophenetic coefficient was calculated. We selected the cluster number closest to a cophenetic coefficient of 0.95. Cell states less than 10 marker genes and those likely to be false positives according to false-positive index (AFI) were removed from our analysis^28^.

In order to recovering cell states in external datasets, EcoTyper implements the NMF model to devise a reference-based strategy. In brief, EcoTyper takes advantage of NMF to apply the model learned in the discovery cohorts to validation cohorts. This method has three advantages over other supervised classification. First, the mathematical structure of the original model is maintained when classifying the samples in the new dataset, which can avoid the biasws caused by training the new classifier. Second, this approach is based on the original model and ensures consistent interpretation. Third, different from the method of independently performing supervised classification on each sample, the matrix H’ is jointly updated on all samples, which is conducive to increasing the robustness of cell state recovery.

### UMAP visualization of cell states

To visualize the cell states in 12 cell types, we used the umap package in R with default parameters^45^. The imputed cell type-specific gene expression profiles were subjected to EcoTyper and the Euclidean distance was used in this analysis. This was done for CRC patients that assigned to cell states. A Uniform Manifold Approximation and Projection (UMAP) embedding was created for each cell type in CRC.

### Cell states recovery in validation CRC cohorts

EcoTyper implements the NMF model to devise a reference-based strategy for recovering predefined cell states in external datasets^9^. We used EcoTyper to apply model learnt in the discovery cohorts to validation cohorts of CRC. For each cell type, the cell state recovery model in EcoTyper generated a coefficient matrix based on gene expression matrix, where each cell state was represented as a weight. Permutation testing was used to statistically evaluate the recovery of individual cell states in the validation cohorts of CRC patients, the statistical confidence was determined with the z-score. Moreover, z-scores >1.65 and one-sided *p*-value < 0.05 were considered significant. This framework was used in validation cohorts and scRNA-seq data of CRC patients.

### Identification of ecotypes in CRC patients

We used EcoTyper to identify ecotypes in CRC patients, which implements a multicellular community’s identification algorithm that maximizes the pairing co-association between cell states while minimizing mutual avoidance within the cluster^9^. In brief, we first quantified the cell states as discrete variables, in which each patient was assigned to the most abundant cell state per cell type. Next, we generated a binary matrix ${{{{{\rm{A}}}}}}$ with cell states as rows and CRC patients as columns. The entries in ${{{{{\rm{A}}}}}}$ were defined as follows:

i $${A}_{ij}=\left\{\begin{array}{c}1,\,{if cell}\,{state}\,i\,{is}\,{assigned}\,{to}\,{patient}\,j\\ 0,\,{otherwise}\end{array}\right.$$

A Jaccard matrix was generated by calculating all pairwise combinations of cell states. A hypergeometric test was performed for each pair of cell states under the null hypothesis of no overlap of two cell states. The Jaccard indices were set to 1 if *P*-value was greater than 0.01 and 0 for other conditions. Finally, the ‘hclust’ function in R package stats was used to conduct unsupervised clustering of Jaccard matrix. The optimal number of clusters was determined based on silhouette width maximization. The network diagrams of CRC ecotypes networks were generated in Cytoscape^46^, and the edge thickness represents Jaccard index between cell states that assigned to each sample.

### Survival analysis of cell states and ecotypes

To determine the clinical relevance of cell states and ecotypes in CRC, we performed the survival analysis based on the ‘survfit’ function from survival package^47^. The survival times of patients with one cell states or ecotypes were compared with others. The cox proportional hazards model was used to determine the risky and protective cell states and ecotypes in CRC. *P*-values less than 0.05 were considered to be significant.

### Functional analysis of cell states and ecotypes

To predict the functions of cell states and ecotypes, we first identified the marker genes in each cell state and ecotype with EcoTyper. Next, we used clusterProfiler to perform functional enrichment analysis^48^. The biological processes in Gene ontology (GO) were considered in our analysis. We only considered the GO terms with genes ranged from 15 to 500. The biological processes with *P* < 0.01 and adjusted *P* < 0.05 were considered to be significant. Top ten biological functions with the highest gene counts in each cell state were selected for visualization. We performed the same analysis for ecotypes in CRC. Moreover, we downloaded the cancer hallmark pathways from MsigDB^49^ and the immune-related pathways were obtained from ImmPort project^35,50^. Hypergeometric test was used to evaluate whether the marker genes of a cell state or ecotype were enriched in cancer-related pathways. Pathways with adjusted *P* < 0.05 were considered to be significant.

Moreover, we performed the Gene Set Enrichment Analysis (GSEA) to identify the functional pathways enriched by genes highly expressed in each cell state^51^. The CRC patients were first divided into two groups based on the cell state, within and without cell state *i*. A rank score was calculated for each gene as follow:

ii $${{{{{\rm{S}}}}}}\left({{{{{{\rm{g}}}}}}}_{j}\right)=-{{\log }}\left({{{{{\rm{p}}}}}}\right)* {{{{{\rm{sign}}}}}}({{\log }}({{{{{\rm{FC}}}}}}))$$

Where *p* was the *p*-value for Wilcoxon’s rank sum test between two groups and FC was the fold change between the expressions of patients within and without cell state *i*. Next, all genes were subjected to GSEA and the enriched functional pathways were identified.

### Transcriptional regulators of ecotypes in CRC

To identify the transcription factors (TFs) that potentially target the genes highly expressed in CEs, we used the RcisTarget to perform the enrichment analysis^52^. We first identified the highly expressed genes in each CE by Wilcoxon’s rank sum test. Genes with adjusted *p*-values less than 0.05 were identified and subjected into RcisTarget analysis. The TF binding sites upstream 500 bp of transcription start sites were used and the ‘cistarget’ function was used with the parameters (nesThreshol = 3, AUC = 3*sd(auc) + mean(auc)). The enriched motifs were ranked based on the normalized enrichment score (NES) and top two were visualized for each CE in CRC.

### Therapeutic benefits of cell states and ecotypes in CRC

To investigate the therapeutic benefit of the drug for each cell state and ecotype, we used ‘survifit’ function in survival package to analyze the drug therapy response data in CRC (GSE72970)^53,54^. Two first-line chemotherapy treatments for CRC patients were analyzed, including FOLFIRI and FOLFOX. The differences in survival times between responders and non-responders in each cellular state and ecotype were compared. If the survival times of responders were significantly longer than those of non-responders, it indicated that the cell state or ecotype can benefit from treatments. The FOLFIRI and FOLFOX treatments were analyzed separately.

Moreover, we predicted clinical drug response in TCGA CRC patients based on cell line drug screening datasets from Genomics of Drug Sensitivity in Cancer (GDSC)^55,56^. Combat was used for batch effects correction^40^ and 20% genes with low variances were excluded from this analysis. In total, 198 drug responses were predicted for all CRC patients. Patients were divided into sensitive and resistant groups based on the median of drug AUC. The survival package in R was used to evaluate the differences in survival between two groups.

### Statistics and reproducibility

In total, 1725 CRC patients from 14 independent public datasets (ranged from 14 to 585) were collected from Gene Expression Omnibus (GEO) and The Cancer Genome Atlas (TCGA, *n* = 625). And 37,771 and 15,366 high-quality cells were obtained from GSE132465 and E-MTAB-8107, respectively. The statistical significance of differences between groups was evaluated using Wilcoxon’s rank sum test. The survival difference between groups was assessed by log-rank test. The statistical analyses were performed using Rstudio with R software. *P* value < 0.05 was considered statistically significant. Source data were provided as supplementary table.

### Reporting summary

Further information on research design is available in the Nature Portfolio Reporting Summary linked to this article.

## Supplementary information

Supplementary Information

Description of Additional Supplementary Files

Supplementary Data 1

Supplementary Data 2

Supplementary Data 3

Supplementary Data 4

Supplementary Data 5

Supplementary Data 6

Supplementary Data 7

Reporting Summary

## Data Availability

Public gene expression profiles used in this work can be acquired from the TCGA Research Network portal (https://portal.gdc.cancer.gov/) and Gene Expression Omnibus (GEO, http://www.ncbi.nlm.nih.gov/geo/). A list of accessions of publicly available datasets used in this study can also be found in Supplementary Data 1. Source data for figures were provided in Supplementary Data 7. The other datasets used and/or analyzed during the present study are available from the corresponding authors on reasonable request.

## Code availability

The codes of EcoTyper were obtained from https://ecotyper.stanford.edu/carcinoma/. Other codes used in this study are available from https://github.com/ComputationalEpigeneticsLab/Tumor-cellular-states-and-ecosystems-in-CRC and Zenodo^57^.

## References

[1] Ferlay J (2010). Estimates of worldwide burden of cancer in 2008: GLOBOCAN 2008. Int J. Cancer.

[2] Punt CJ, Koopman M, Vermeulen L (2017). From tumour heterogeneity to advances in precision treatment of colorectal cancer. Nat. Rev. Clin. Oncol..

[3] Ciardiello D (2019). Immunotherapy of colorectal cancer: challenges for therapeutic efficacy. Cancer Treat. Rev..

[4] Patel SA, Minn AJ (2018). Combination cancer therapy with immune checkpoint blockade: mechanisms and strategies. Immunity.

[5] Le DT (2015). PD-1 blockade in tumors with mismatch-repair deficiency. N. Engl. J. Med..

[6] Thorsson V (2018). The immune landscape of cancer. Immunity.

[7] Wang H (2022). Subtyping of microsatellite stability colorectal cancer reveals guanylate binding protein 2 (GBP2) as a potential immunotherapeutic target. J. Immunother. Cancer.

[8] Sadanandam A (2013). A colorectal cancer classification system that associates cellular phenotype and responses to therapy. Nat. Med..

[9] Luca BA (2021). Atlas of clinically distinct cell states and ecosystems across human solid tumors. Cell.

[10] Suva ML, Tirosh I (2019). Single-cell RNA sequencing in cancer: lessons learned and emerging challenges. Mol. Cell.

[11] Zhang L (2020). Single-cell analyses inform mechanisms of myeloid-targeted therapies in colon cancer. Cell.

[12] Mei Y (2021). Single-cell analyses reveal suppressive tumor microenvironment of human colorectal cancer. Clin. Transl. Med..

[13] Kim M (2021). Single-cell RNA sequencing reveals distinct cellular factors for response to immunotherapy targeting CD73 and PD-1 in colorectal cancer. J. Immunother. Cancer.

[14] Li H (2017). Reference component analysis of single-cell transcriptomes elucidates cellular heterogeneity in human colorectal tumors. Nat. Genet.

[15] Lee HO (2020). Lineage-dependent gene expression programs influence the immune landscape of colorectal cancer. Nat. Genet.

[16] Qian J (2020). A pan-cancer blueprint of the heterogeneous tumor microenvironment revealed by single-cell profiling. Cell Res..

[17] Nishina T (2021). Interleukin-11-expressing fibroblasts have a unique gene signature correlated with poor prognosis of colorectal cancer. Nat. Commun..

[18] Zhou Y (2020). Single-cell multiomics sequencing reveals prevalent genomic alterations in tumor stromal cells of human colorectal cancer. Cancer Cell.

[19] Zheng H, Liu H, Ge Y, Wang X (2021). Integrated single-cell and bulk RNA sequencing analysis identifies a cancer associated fibroblast-related signature for predicting prognosis and therapeutic responses in colorectal cancer. Cancer Cell Int..

[20] Deng L, Jiang N, Zeng J, Wang Y, Cui H (2021). The versatile roles of cancer-associated fibroblasts in colorectal cancer and therapeutic implications. Front Cell Dev. Biol..

[21] Huang H (2018). High expression of COL10A1 is associated with poor prognosis in colorectal cancer. Onco Targets Ther..

[22] Wei C (2019). Crosstalk between cancer cells and tumor associated macrophages is required for mesenchymal circulating tumor cell-mediated colorectal cancer metastasis. Mol. Cancer.

[23] Wang X (2016). THBS2 is a potential prognostic biomarker in colorectal cancer. Sci. Rep..

[24] Deng B, Liu XP, Wang X (2021). Prognostic and immunological role of THBS2 in colorectal cancer. Biomed. Res. Int..

[25] Liang L (2022). ‘Reverse Warburg effect’ of cancerassociated fibroblasts (Review). Int J. Oncol..

[26] Liu Z (2022). Machine learning-based integration develops an immune-derived lncRNA signature for improving outcomes in colorectal cancer. Nat. Commun..

[27] Pi YN, Guo JN, Lou G, Cui BB (2021). Comprehensive analysis of prognostic immune-related genes and drug sensitivity in cervical cancer. Cancer Cell Int..

[28] Steen CB (2021). The landscape of tumor cell states and ecosystems in diffuse large B cell lymphoma. Cancer Cell.

[29] Xu X (2021). IRF1 regulates the progression of colorectal cancer via interferoninduced proteins. Int J. Mol. Med..

[30] Yang Y (2020). FOXM1/DVL2/Snail axis drives metastasis and chemoresistance of colorectal cancer. Aging.

[31] Liu Y (2021). Author Correction: TP53 loss creates therapeutic vulnerability in colorectal cancer. Nature.

[32] Allen JD (2012). Probe mapping across multiple microarray platforms. Brief. Bioinform.

[33] Stalteri MA, Harrison AP (2007). Interpretation of multiple probe sets mapping to the same gene in Affymetrix GeneChips. BMC Bioinforma..

[34] Gupta S, Verma AK, Ahmad S (2020). Feature selection for topological proximity prediction of single-cell transcriptomic profiles in drosophila embryo using genetic algorithm. Genes.

[35] Li Y (2020). Pan-cancer characterization of immune-related lncRNAs identifies potential oncogenic biomarkers. Nat. Commun..

[36] Jiang T (2021). ImmReg: the regulon atlas of immune-related pathways across cancer types. Nucleic Acids Res..

[37] Xu J (2020). MIR22HG acts as a tumor suppressor via TGFbeta/SMAD signaling and facilitates immunotherapy in colorectal cancer. Mol. Cancer.

[38] Gautier L, Cope L, Bolstad BM, Irizarry RA (2004). affy-analysis of Affymetrix GeneChip data at the probe level. Bioinformatics.

[39] Leek JT, Johnson WE, Parker HS, Jaffe AE, Storey JD (2012). The sva package for removing batch effects and other unwanted variation in high-throughput experiments. Bioinformatics.

[40] Johnson WE, Li C, Rabinovic A (2007). Adjusting batch effects in microarray expression data using empirical Bayes methods. Biostatistics.

[41] Butler A, Hoffman P, Smibert P, Papalexi E, Satija R (2018). Integrating single-cell transcriptomic data across different conditions, technologies, and species. Nat. Biotechnol..

[42] Aran D (2019). Reference-based analysis of lung single-cell sequencing reveals a transitional profibrotic macrophage. Nat. Immunol..

[43] Newman AM (2019). Determining cell type abundance and expression from bulk tissues with digital cytometry. Nat. Biotechnol..

[44] Newman AM (2015). Robust enumeration of cell subsets from tissue expression profiles. Nat. Methods.

[45] McInnes L., Healy J., Melville J. Umap: Uniform manifold approximation and projection for dimension reduction. Preprint at https://arxiv.org/abs/1802.03426 (2018).

[46] Shannon P (2003). Cytoscape: a software environment for integrated models of biomolecular interaction networks. Genome Res..

[47] Terry M. Therneau, P. M. G. *Modeling Survival Data: Extending the Cox Model* (Springer, 2000).

[48] Wu T (2021). clusterProfiler 4.0: a universal enrichment tool for interpreting omics data. Innovation.

[49] Liberzon A (2015). The Molecular Signatures Database (MSigDB) hallmark gene set collection. Cell Syst..

[50] Bhattacharya S (2014). ImmPort: disseminating data to the public for the future of immunology. Immunol. Res..

[51] Subramanian A (2005). Gene set enrichment analysis: a knowledge-based approach for interpreting genome-wide expression profiles. Proc. Natl Acad. Sci. USA.

[52] Van de Sande B (2020). A scalable SCENIC workflow for single-cell gene regulatory network analysis. Nat. Protoc..

[53] Del Rio M (2017). Molecular subtypes of metastatic colorectal cancer are associated with patient response to irinotecan-based therapies. Eur. J. Cancer.

[54] Cherradi S, Martineau P, Gongora C, Del Rio M (2019). Claudin gene expression profiles and clinical value in colorectal tumors classified according to their molecular subtype. Cancer Manag Res..

[55] Yang W (2013). Genomics of Drug Sensitivity in Cancer (GDSC): a resource for therapeutic biomarker discovery in cancer cells. Nucleic Acids Res..

[56] Maeser D, Gruener RF, Huang RS (2021). oncoPredict: an R package for predicting in vivo or cancer patient drug response and biomarkers from cell line screening data. Brief. Bioinform.

[57] Li, S. et al. R code for: deep immunophenotyping reveals clinically distinct cellular states and ecosystems in large-scale colorectal cancer. *Zenodo*10.5281/zenodo.8119162 (2023). 10.1038/s42003-023-05117-1 PMC10374645 37500893

