# Supplementary material for: Deep immunophenotyping reveals clinically distinct cellular states and ecosystems in large-scale colorectal cancer
Source: Commun Biol. 2023 Jul 27;6:785. doi: 10.1038/s42003-023-05117-1 (PMC10374645; doi:10.1038/s42003-023-05117-1)
Supplement: Supplementary file 1 — Supplementary Information [file 42003_2023_5117_MOESM1_ESM.pdf]

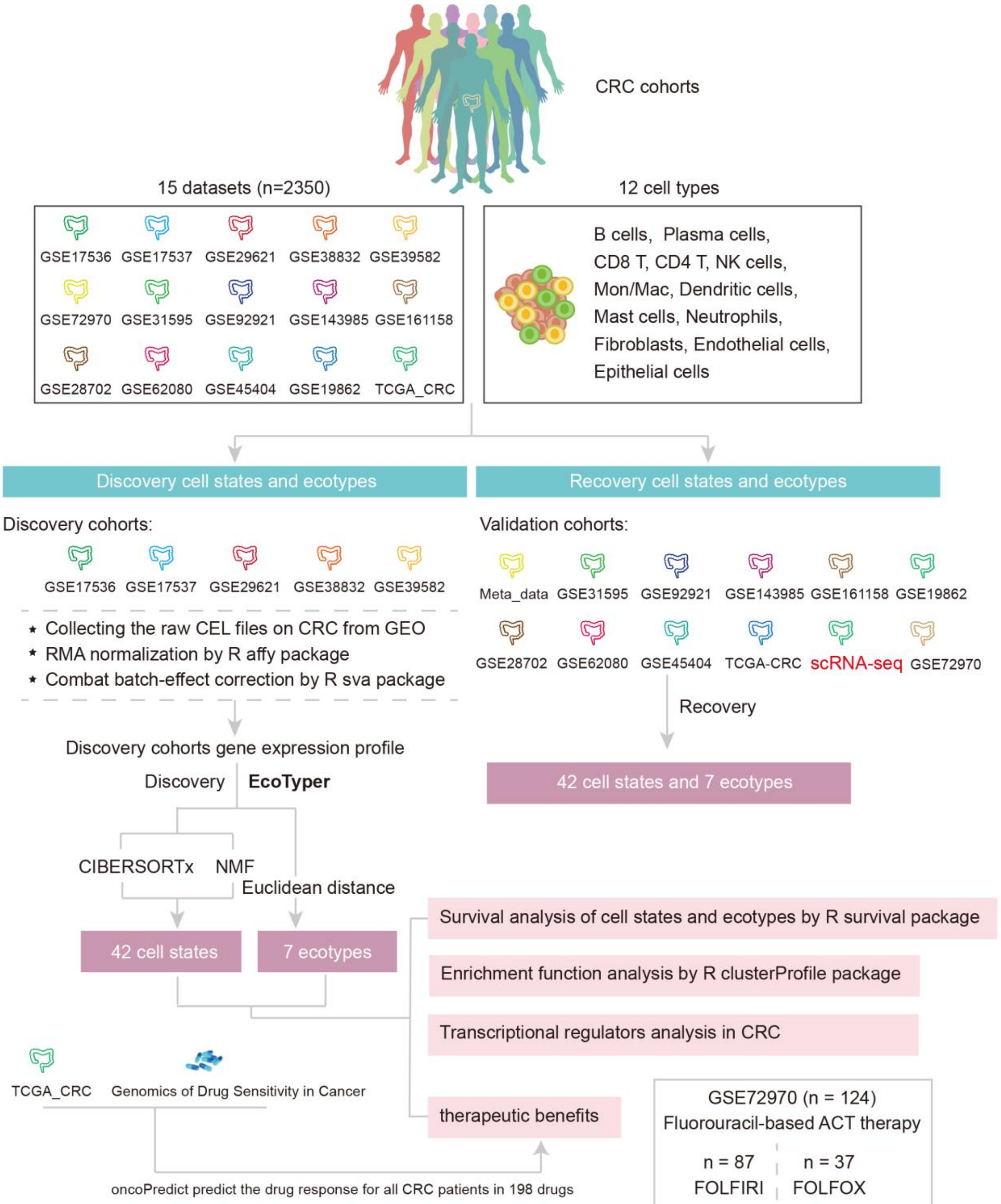

Supplementary Fig. 1. Overview of the framework for characterization of the cellular states and ecotypes in CRC.

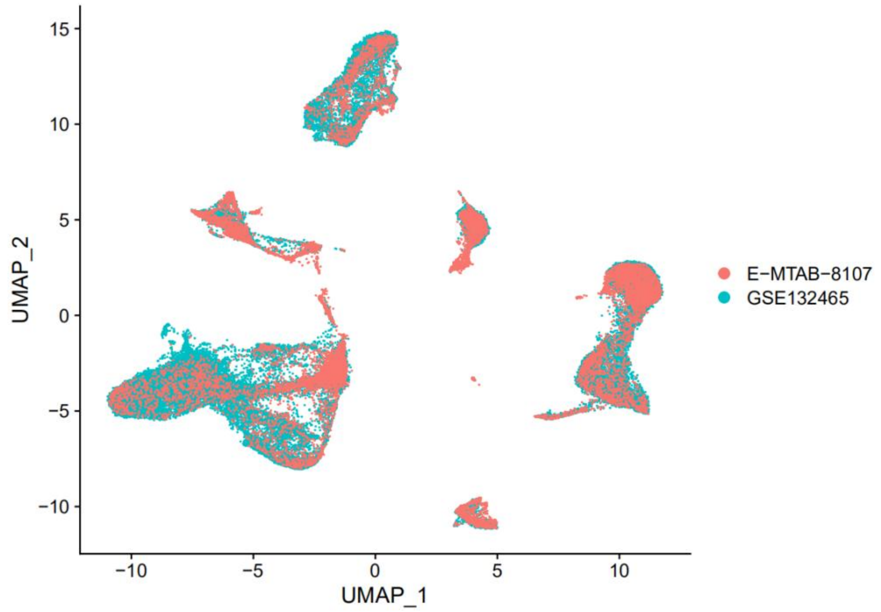

Supplementary Fig. 2. UMAP plot showing the cells in single cell transcriptome. Cells were colored by study.

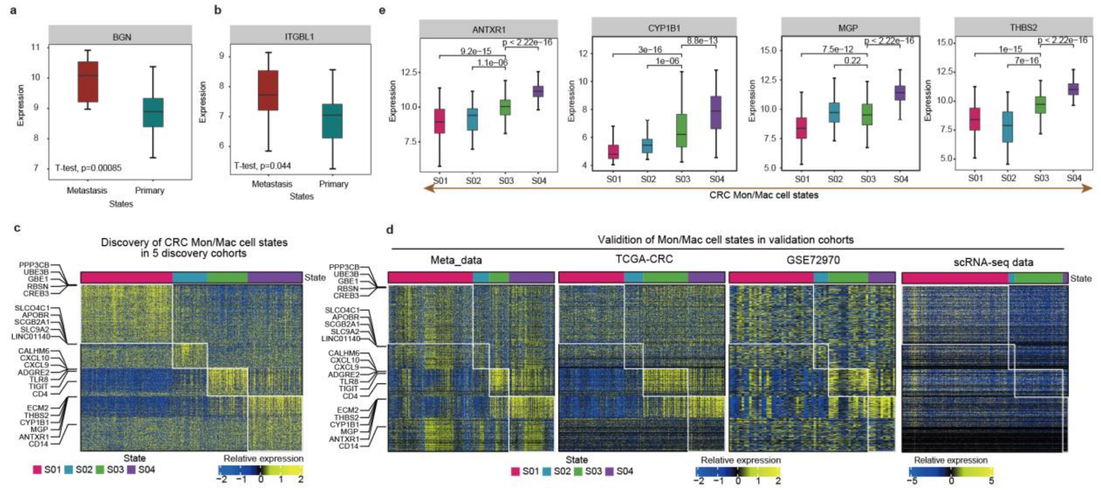

Supplementary Fig. 3. Landscape of cellular states in CRC. a Boxplot showing the expressions of BGN in primary and metastasis CRC patients. **b** Boxplot showing the expressions of ITGBL1 in primary and metastasis CRC patients. The lines in each box plot represent median values, and the box limits represent upper and lower quantiles. **c** Heatmap depicting four Mon/Mac cell states identified from CRC bulk transcriptomes of discovery cohorts. Patient samples are organized by the most prevalent cell states and genes used for discovery of the cell states are shown. **d** Heatmap depicting four Mon/Mac cell states in validation cohorts and single cell transcriptome. **e** Boxplots showing the expressions of marker genes in four states of Mon/Mac cellular states, including ANTXR1, CYP1B1, MGP and THBS2. The lines in each box plot represent median values, and the box limits represent upper and lower quantiles.

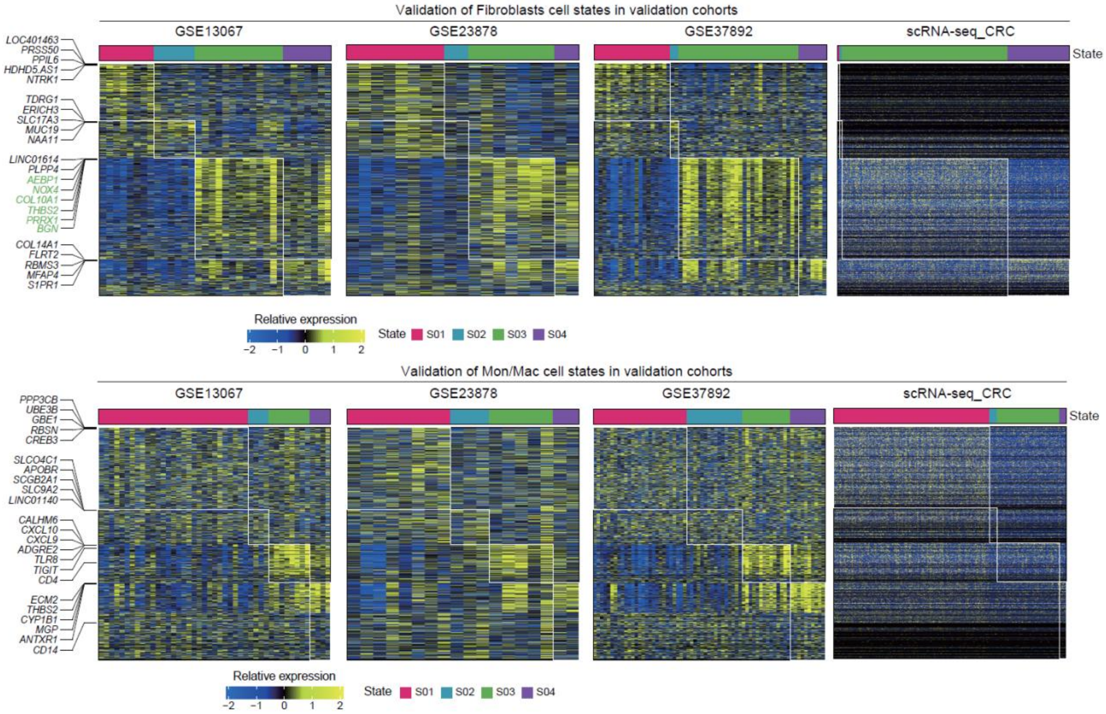

Supplementary Fig. 4. Validation of cell states in another four validation CRC cohorts.

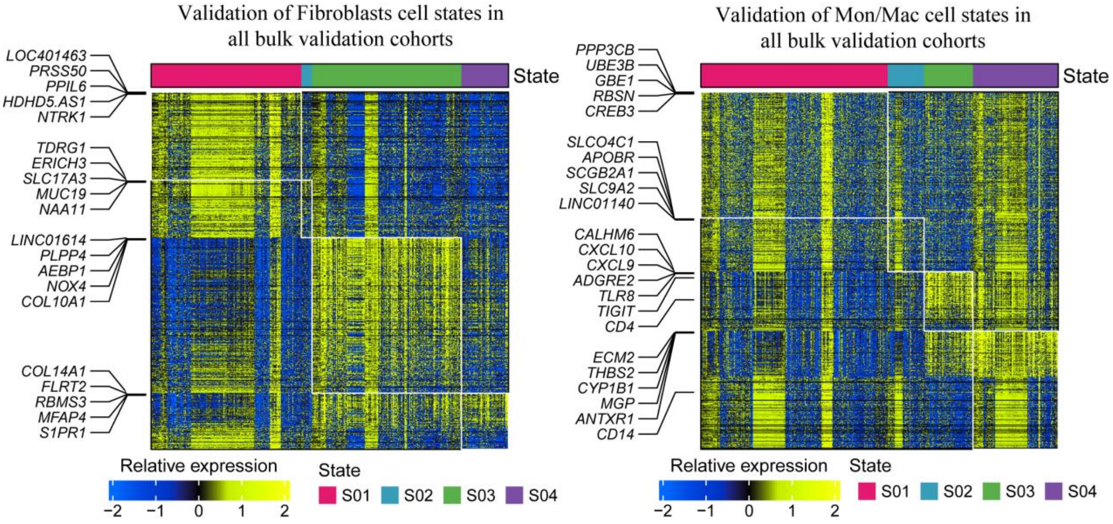

Supplementary Fig. 5. Validation of cell states in combined validation bulk CRC transcriptomes.

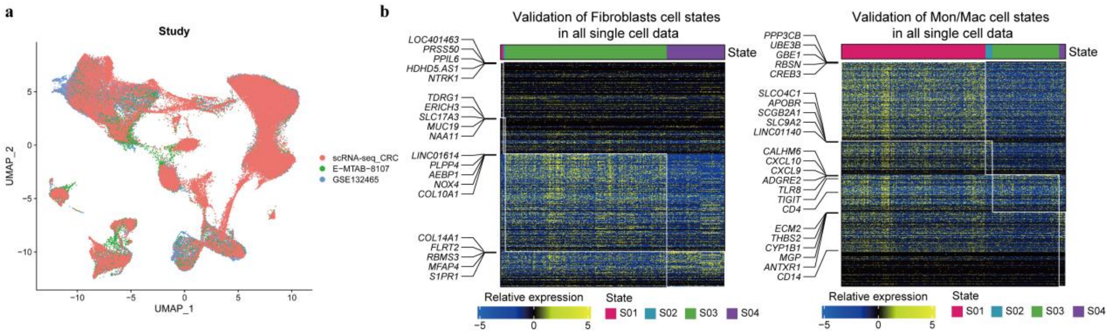

Supplementary Fig. 6. Validation of cell states in combined validation single-cell

CRC transcriptomes. a UMAP plot showing the cells in single cell transcriptome. Cells were colored by study. **b** Validation of cell states.

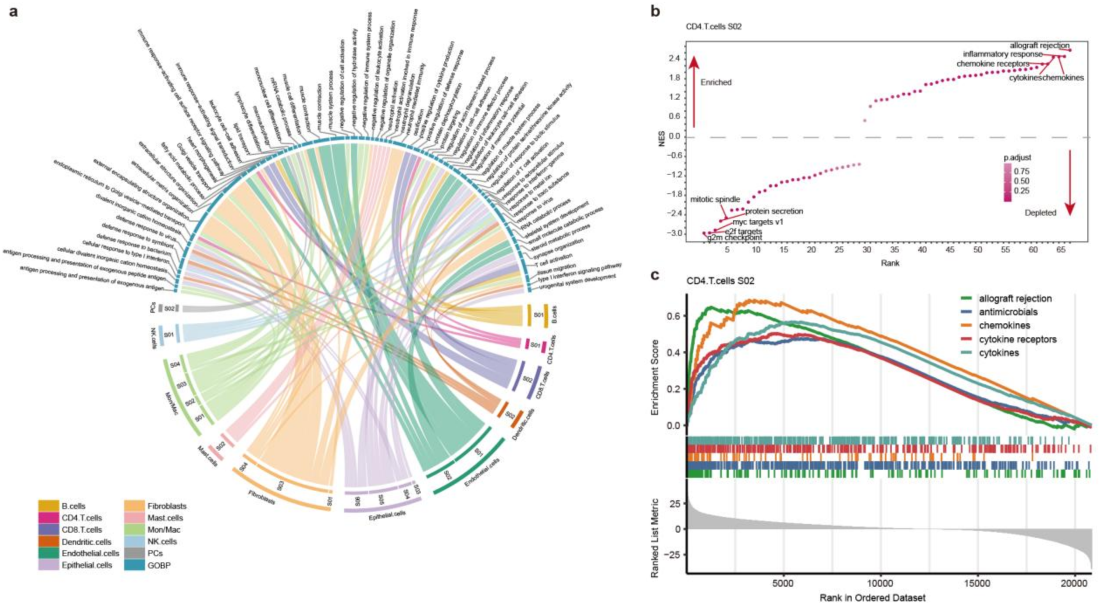

Supplementary Fig. 7. Functional profiling of cellular states in CRC. a Circos plot depicting the enriched biological processes for genes highly expressed in cellular states in CRC. **b** Scatter plot showing the normalized enrichment scores (NES) of pathways in CD4 T cells S02 cell states. Pathways are ranked by NES. Colors of dots are corresponding to the p-values. **c** The enrichment score (ES) distribution for the genes differentially expressed in CD4 T cells S02 enriched in five pathways. Each line is for a pathway.

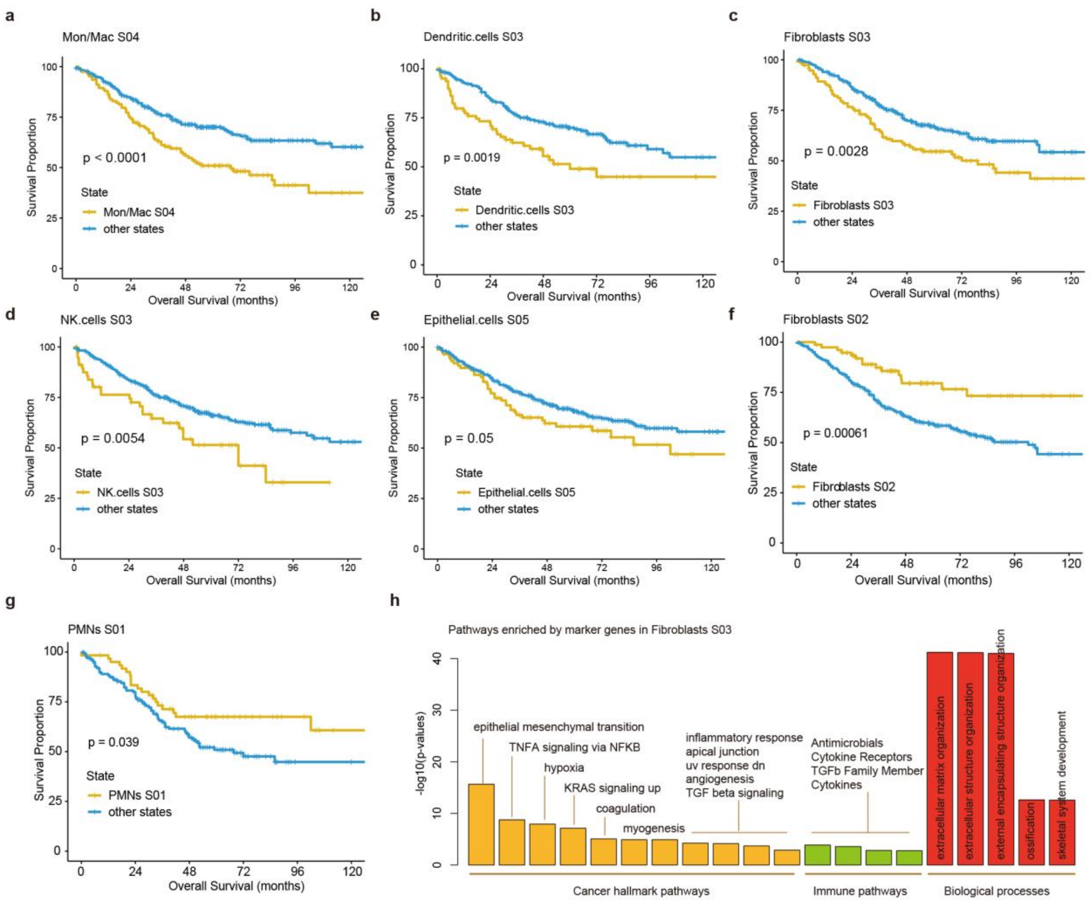

Supplementary Fig. 8. Cellular state associated with patient survival in CRC. a-g Kaplan-Meier plots showing differences in overall survival between patients within specific cellular states and other states in discovery cohorts. **h** Barplots showing the enriched functional pathways for genes highly expressed in fibroblasts S03.

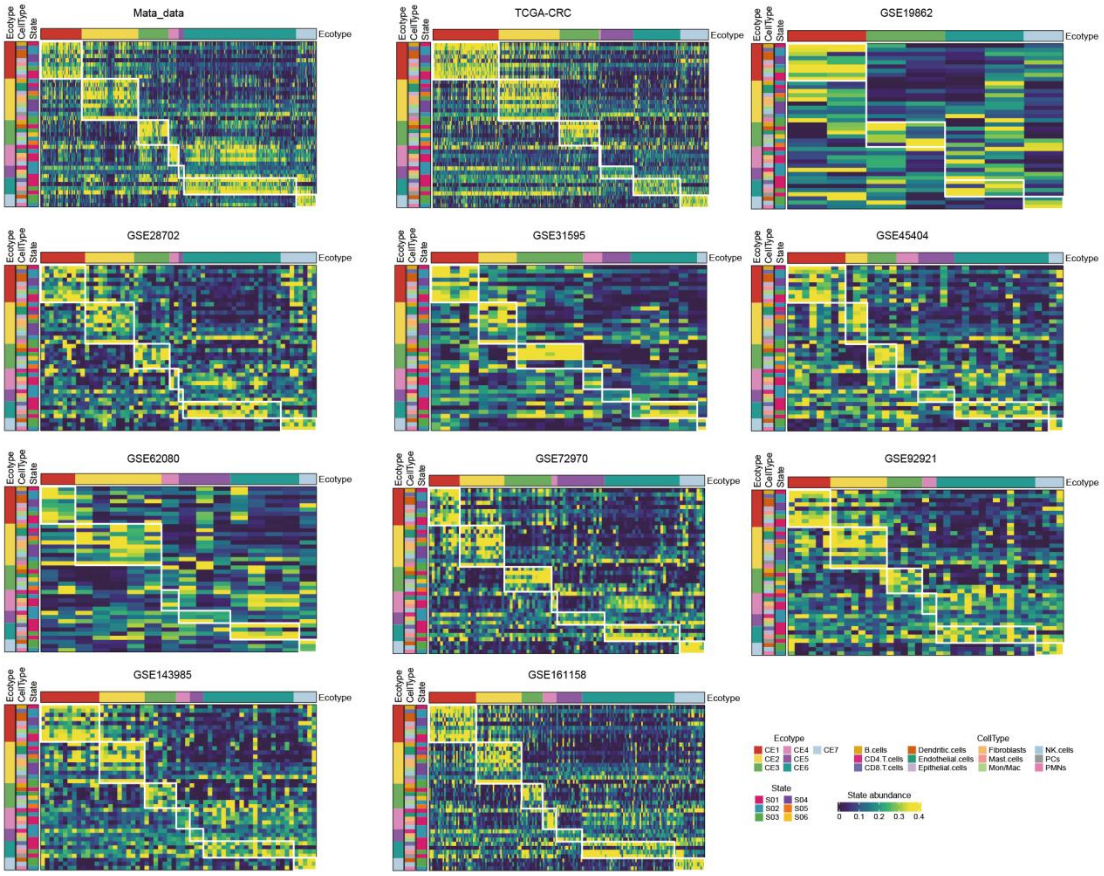

Supplementary Fig. 9. Validation of the ecotypes in CRC. Cell state abundance patterns in the CRC validation cohorts, with cell states organized into seven colorectal ecotypes (CEs) and tumor samples (columns) ordered by the most abundant CE per sample. Each panel is for one validation cohort.

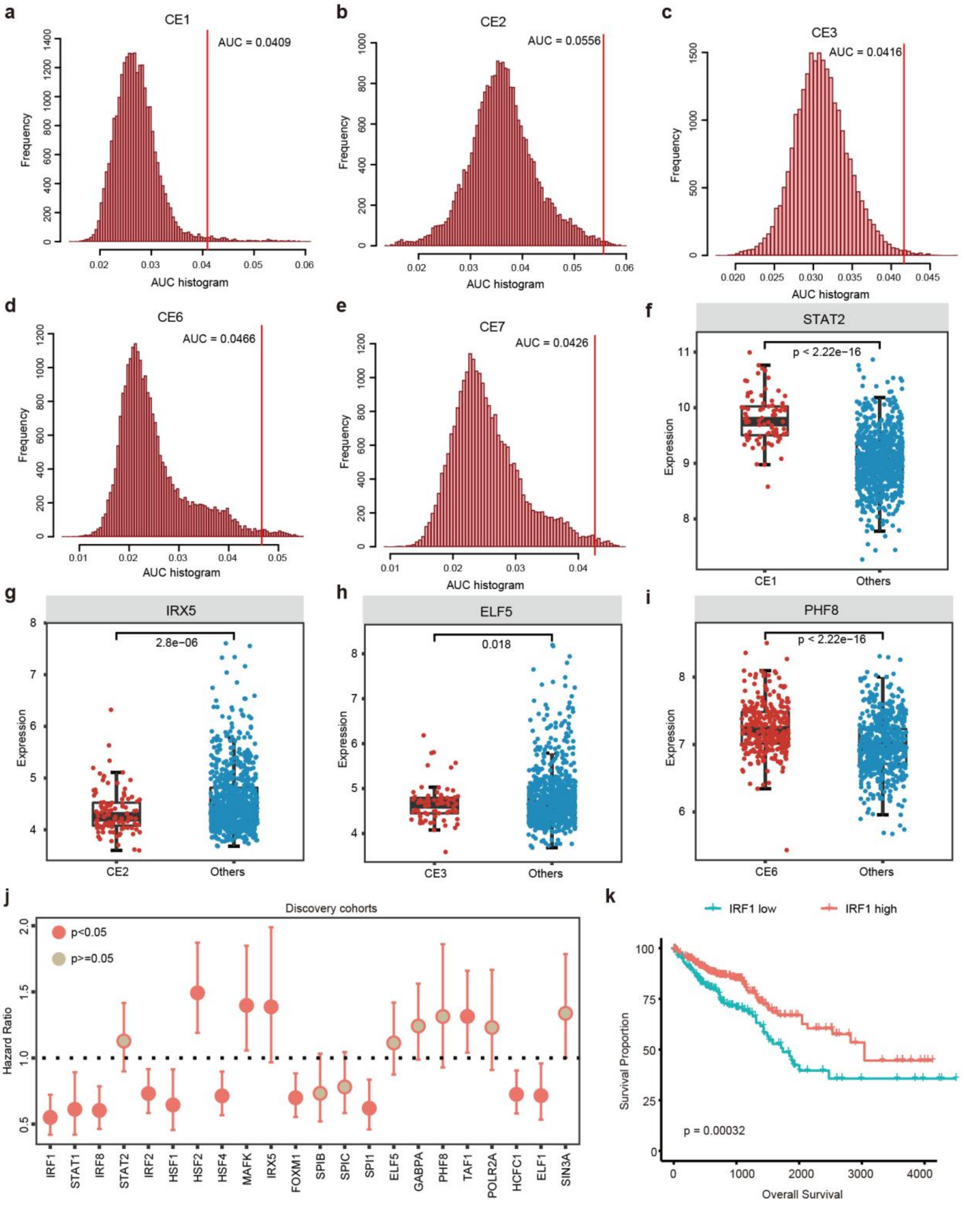

Supplementary Fig. 10. Transcriptional regulators of cellular states in CRC. a-e Barplots showing the distribution of AUCs and the corresponding threshold used for identifying the TFs in each CE. **f-i** Boxplots showing the expressions of TFs in CEs of CRC. **f** for STAT2, **g** for IRX5, **h** for ELF5 and **i** for PHF8. The lines in each box plot represent median values, and the box limits represent upper and lower quantiles. **j** The hazard ratios (HRs) and 95% confidence levels for the expressions of TFs associated with clinical survival in discovery cohorts. Centre point is the HR, and bounds are the 95% confidence levels. **k** Kaplan-Meier plots showing differences in survival between patients with high and low expression of IRF1.

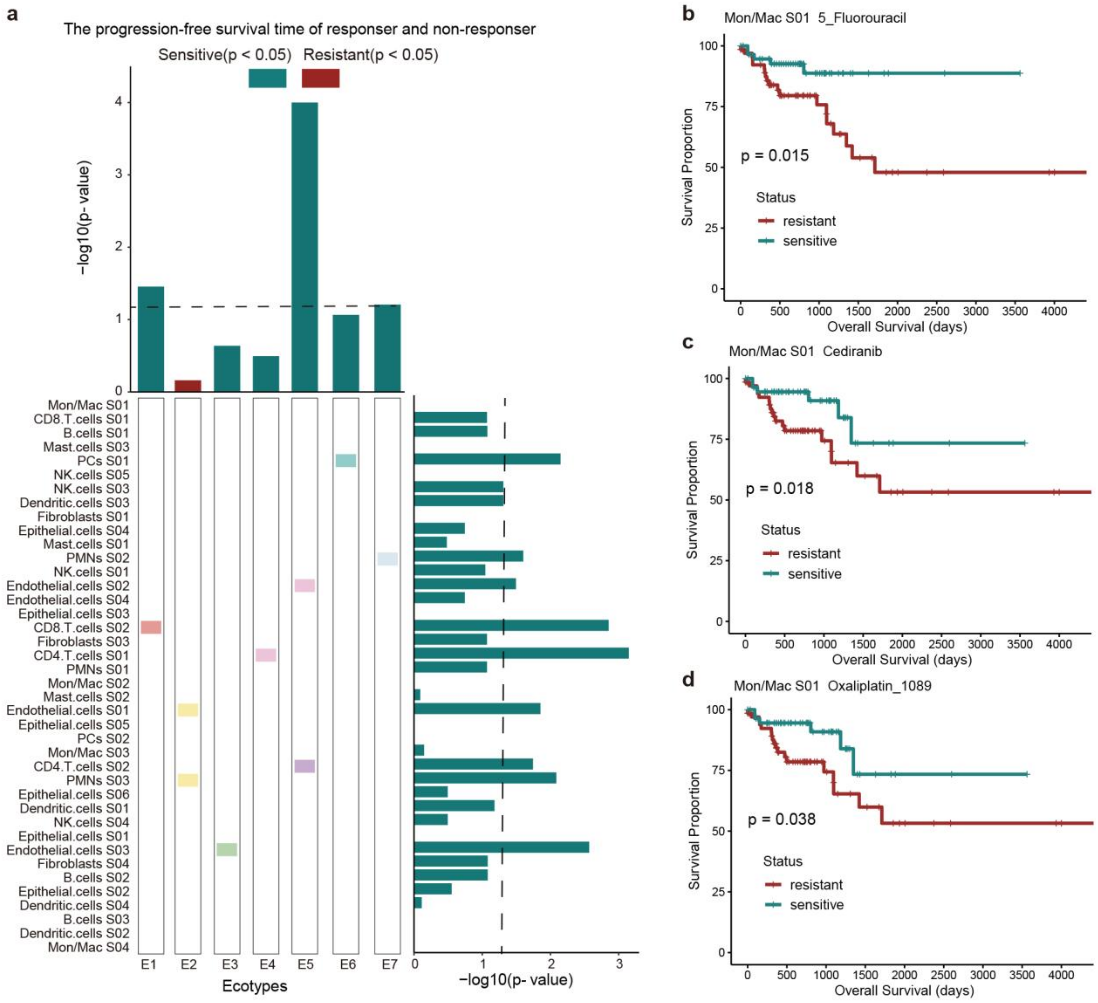

Supplementary Fig. 11. Cellular states associated with drug treatment response in CRC. a Association between cellular states and therapeutic benefit from FOLFIRI and FOLFOX treatments in CRC. Cell states were ranked by p-values of the associations with progression-free survival. **b-d** Kaplan-Meier plots showing differences in survival between responders and non-responders with Mon/Mac S01 states. **b** for 5-fluorouracil treatment, **c** for cediranib treatment and **d** for oxaliplatin treatment.

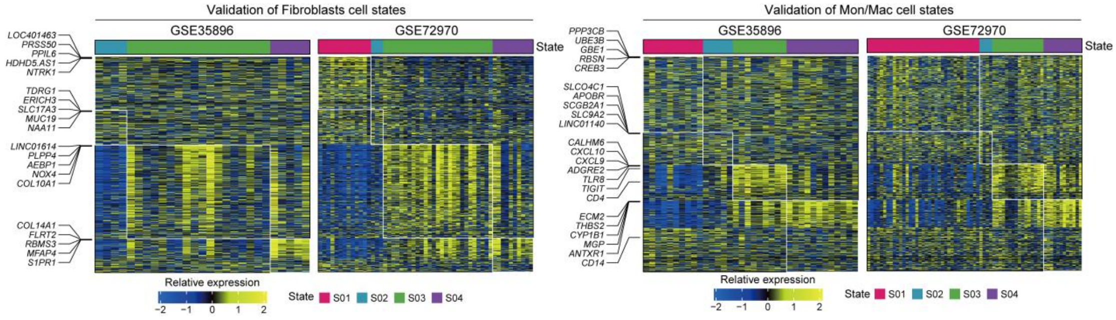

Supplementary Fig. 12. Validation of cell states in a new validation cohort and a previous validation cohort. For multiple probes mapping to the same gene the maximum value was consider.
