# Supplementary material for: Deep immunophenotyping reveals clinically distinct cellular states and ecosystems in large-scale colorectal cancer
Source: Commun Biol. 2023 Jul 27;6:785. doi: 10.1038/s42003-023-05117-1 (PMC10374645; doi:10.1038/s42003-023-05117-1)
Supplement: Supplementary file 2 — Description of Additional Supplementary Files [file 42003_2023_5117_MOESM2_ESM.pdf]

Description of Additional Supplementary Files

File name: Supplementary Data 1
Description: The details of colorectal cancer datasets.

File name: Supplementary Data 2
Description: The recovery of cell states in independent CRC cohorts.

File name: Supplementary Data 3
Description: The enriched functional pathways of marker genes in cellular states of CRC.

File name: Supplementary Data 4
Description: The survival associations of all cell states.

File name: Supplementary Data 5
Description: The identification of seven colorectal ecotypes in discovery cohorts.

File name: Supplementary Data 6
Description: The cellular states predict therapeutic benefits for a large number of drugs in CRC.

File name: Supplementary Data 7
Description: The source data for figures.
